# The Impact of Policy Interventions on Systemic Risk across Banks

**DOI:** 10.1007/s10693-023-00404-8

**Published:** 2023-02-22

**Authors:** Simona Nistor, Steven Ongena

**Affiliations:** 1 Faculty of Economics and Business Administration, Babeş-Bolyai University of Cluj-Napoca, Teodor Mihali Street, Nr. 58-60, 400591 Cluj-Napoca, Romania; 2 University of Zürich, Swiss Finance Institute, KU Leuven, NTNU Business School and CEPR, Plattenstrasse 14, 8032 Zürich, Switzerland

**Keywords:** Systemic risk, Policy interventions, Risk strategies, Regulatory restrictions, E58, G01, G21, G28, H81

## Abstract

What is the impact of policy interventions on the systemic risk of banks? To answer this question, we analyze a comprehensive sample that combines an original set of bank-specific bailout events with the balance sheets of key affected and nonaffected European banks between 2005 and 2014. We find a positive and significant association of *guarantees* with systemic risk that is somewhat weaker in the long run when the regulator appoints members to the supervisory board. The short run association between *recapitalizations* and systemic risk is also positive for large and less capitalized banks, while in the long run, recapitalizations are linked with reduced systemic importance, especially for less profitable banks and in cases when the regulator limits management pay. *Liquidity injections* are positively linked with systemic risk, but the long-run effect is mitigated for small or better capitalized banks. In the short run, injecting liquidity is associated with reduced systemic risk when the regulator imposes restrictions on supervisory board composition or on management pay or capital payouts.

**Supplementary Information:**

The online version contains supplementary material available at 10.1007/s10693-023-00404-8.

## Introduction

The recent financial crisis led to coordinated efforts by governments and central banks to avoid a major systemic meltdown. Public interventions such as debt guarantees, capital injections, state loans, acquisitions of impaired assets, and/or nationalizations were implemented on an unprecedented scale by most countries.^1^ At the European Union (EU) level, policy actions adopted by member states immediately after the Lehman Brothers collapse were coordinated in a massive bailout of financial institutions that was estimated to have amounted to 3.65 trillion euro (European Commission 2009).^2^ Since then, several additional financial support programs have been set up, especially after the European sovereign debt crisis and the Greek bailout in 2010.^3^ These types of emergency assistance programs play an important role in restoring public confidence in the banking sector. However, how effective are these different tools in controlling systemic risk, and how heterogeneous is their impact across banks’ risk strategies? Given the current COVID-19 pandemic and its potential dire economic and financial consequences for the banking sector, we think this is a highly relevant policy question.

Our paper contributes to this literature by investigating the interplay between systemic risk, policy interventions, regulatory restrictions, and banks’ risk strategies. Our study analyses 83 key global and local financial institutions from 22 European countries during the 2005–2014 period (see Online Appendix 1). First, we are interested in assessing the immediate effects of policy interventions on systemic risk. Second, we examine their long-run implications. While in the short run regulators aim to restore confidence and alleviate the spread of contagion through interventions, over a longer period, for the bailed-out banks, the too-big-to-fail protection may be traded off with the costs and restrictions associated with bailouts. Moreover, we explore how regulatory restrictions and banks’ risk strategies exacerbate or mitigate the relation between rescue actions and systemic risk. The main research questions that we aim to answer therefore are as follows: *What are the immediate effects of policy interventions on banks’ contribution to systemic risk? How do government interventions affect systemic risk in the long run?*

Clearly, these elements are interdependent. For example, it is more likely that the government provides bailouts to systemically important banks, which due to their size, higher risk, complexity, and/or interconnectedness can have a sizeable adverse impact on financial stability in case of their failure (Berger et al. 2020; Gerhardt and Vander Vennet 2017; Kick et al. 2016). In our empirical analysis, we aim to methodically account for this interdependence using a Heckman selection procedure. The systemic risk indicators are estimated based on the loss generated by the reduction in the banks’ market capitalization under extreme events. We use the *Marginal Expected Shortfall (MES)* of Acharya et al. (2017), a measure that captures systemic risk realizations, as well as forward-looking risk. The negative spillovers from each bank to the system are defined as their contribution to systemic risk or their systemic importance.^4^

Although during recent years several financial assistance programs have been set up to combat the Eurozone crisis,^5^ we focus on the emergency rescue measures most used by European member states during the global financial crisis (GFC) and the European sovereign debt crisis (ESDC). These public interventions, supported by governments or central banks at the national level and agreed to by the European Commission, consisted of instruments designed to limit systemic risk and the spread of contagion during the financial crises in Europe. Our interest resides in assessing the effectiveness of these most flexible policy interventions in controlling systemic risk. To perform this evaluation, we use a unique dataset of bank-level interventions by national authorities (state guarantees, recapitalizations and liquidity injections) collected from banks’ annual reports, financial statements, websites and the State Aid Register of the European Commission (see Online Appendix 2 and Online Appendix 3).

Our empirical findings establish that bailouts are associated with different evolutions of systemic risk in the short versus long run. We find evidence that in the short run, banks that receive policy interventions are linked with an enhanced contribution to systemic risk and that the effect is strongly significant for state guarantees. We argue that the positive association with systemic contribution might be attributed to the realization of risk captured by the MES measure. Interventions are usually implemented after risk is realized. As our dependent variable reflects both systemic risk realizations and forward-looking risk, the valuation of banks by investors is likely to be reduced before intervention and to not recover for some time after. The delayed success of the interventions may also be partly because European states were not always credible providers of financial assistance during the period analyzed. In the long run, liquidity injections − by providing only temporary relief − are associated with enhanced systemic risk, while recapitalizations somewhat are linked with reduced systemic importance. The findings suggest that banks injected with liquidity remained weak and investors penalized their stock returns, which is reflected in a higher systemic contribution. In turn, recapitalizations fix the problem created by risk realizations. We further provide empirical evidence that the link between policy interventions and systemic risk varies when restrictions such as supervisory board intrusions, management pay limitations, and capital payout bans are imposed and across banks with different risk strategies related to their size, leverage, and profitability.

Based on our estimates, important policy conclusions can be drawn. First, emergency policy interventions should be adequately implemented because their association with systemic risk could be different in the long versus short run. Second, their effectiveness can be significantly influenced both by the restrictions imposed by the regulator throughout the duration of the financial assistance and by the banks’ risk strategies.

The remainder of the paper is organized as follows. Section 2 provides the literature review. Section 3 describes the methodology. Section 4 introduces the sample and the data. Section 5 presents the empirical results. Finally, Section 6 concludes.

## Literature review

### General

This paper connects several strands of literature related to studies assessing risk realizations and research focusing on forward-looking risk. First, our paper is linked to research on policy interventions and risk realizations. A number of studies analyze how regulatory policies can help control systemic risk. Considering several international financial crises, Weiß et al. (2014a), for example, find that global systemic risk is significantly influenced by the characteristics of regulatory regimes, explicit deposit insurance schemes and country-wide (macro-) prudential policy interventions (e.g., Karamysheva and Seregina 2020). Recapitalizations can reduce the systemic contribution of banks (López-Espinosa et al. 2012), while liquidity injections can temper the risk incentives of insolvent institutions (Cordella and Yeyati 2003). Buch et al. (2019) show that European banks that received state aid during the crisis are associated with increased systemic importance. However, the U.S. Troubled Assets Relief Program (TARP), based on injections of preferred equity, significantly reduced contributions to systemic risk, particularly for larger and safer banks (Berger et al. 2020). Anginer et al. (2014b) point to the stabilizing role played by deposit insurance arrangements during stress periods but also criticize their destabilizing role in normal times.

Second, our research is related to an extensive literature exploring the forward-looking nature of risk. Numerous papers investigate the moral hazard embedded in deposit insurance schemes (Demirgüç-Kunt and Detragiache 2002; Demirgüç-Kunt and Huizinga 2004; Gropp et al. 2014), recapitalizations (Kane 1995), or liquidity injections (Acharya and Yorulmazer 2007; Farhi and Tirole 2012). Financial help in general may deteriorate the liquidity situation of banks when regulators cannot and/or do not distinguish between illiquid and insolvent financial institutions (Freixas et al. 1999; Repullo 2005). Moreover, rescue packages provided to large banks may incentivize them to engage in highly risky operations (Mishkin 2006), invest in illiquid assets (Cao and Illing 2008) and take on excessive credit risk (Gropp et al. 2014). Additionally, state guarantees provided to efficient banks can enhance their exposure to systemic events (Myerson 2012). On the other hand, the adverse impact of a deposit insurance scheme on systemic risk may diminish if banks hold higher levels of Tier 1 capital (Bostandzic et al. 2014). Homar (2016) highlights the importance of the amount of interventions, presenting empirical evidence that banks that receive large enough capital injections boost the supply of credit, access supplementary funding and improve their balance sheets. Similarly, Giannetti and Simonov (2013) show that a reasonable level of capital injections helped banks increase lending and stimulate investments during the Japanese banking crisis of the 1990s. In the long run, bailouts can increase investors’ expectations of future bailouts (Bayazitova and Shivdasani 2012), especially when banks are considered too big to fail, too interconnected to fail and/or too many to fail (Acharya and Yorulmazer 2007; Brown and Dinç 2011). This could generate enormous costs for shareholders and taxpayers, impede a recovery, and distort competition. Rescued banks may obtain competitive advantages that increase their market power (Berger and Roman 2015), while sound banks that do not receive intervention may increase their loan rates and reduce depositor risk premiums (Koetter and Noth 2016).

### Contributions

We contribute to the extant literature in several ways. First, our foremost contribution is to assess to what extent policy interventions implemented by governments affect systemic risk. Although the isolated impact of the bailout mechanism has been addressed in several theoretical and empirical studies, in comparison with the approaches in other studies, our specifications include several types of interventions with details regarding their volume and their associated restrictions. A large spectrum of the most important policy interventions made by European member states and implemented at the bank level during 2008–2014 is examined: state guarantees, recapitalizations (capital injections) and liquidity injections. The dataset is collected manually from banks’ annual reports, financial statements, websites, and the State Aid Register of the European Commission. Our empirical approach is also different. First, we are interested in investigating the short-run response of systemic risk to policy interventions; in addition, we examine the long-run implications of this relation. This distinction is particularly important for European countries, as governments were not always perceived as trustworthy providers of financial support, which might lead to a delayed effect of interventions.

Second, we contribute to the literature on the restrictions associated with bailouts. In the long run, a tradeoff between too-big-to-fail (TBTF) protection and the regulatory burdens associated with bailouts might come into play. We employ a hand-collected dataset of regulatory restrictions and assess their impact on the relation between policy interventions and systemic risk. Specifically, we consider the following set of restrictions imposed by regulators on the banks receiving intervention during the duration of bailouts: supervisory board intrusions, management pay limitations, and capital payout bans.

Third, we add to the literature by examining the interaction between policy interventions and banks’ risk strategies. We explore how banks’ risk postures related to size, leverage, and profitability exacerbate or mitigate the relation between rescue actions and systemic risk. Especially in the long run, banks are more likely to manifest moral hazard behavior by adjusting their risk strategies.

## Methodology

This section presents the regression specifications used to analyze the impact of the emergency policy interventions made by European national supervisory authorities during the global financial crisis and the European sovereign debt crisis on systemic risk. First, we assess the effects of policy interventions on systemic risk in the short run and in the long run. Second, we explore how banks’ regulatory restrictions and risk strategies exacerbate or mitigate the relation between rescue actions and systemic risk. We employ the Heckman selection approach and control for a variety of bank, market, and macro characteristics, using a sample of 83 publicly listed European banks, with data spanning from 2005 to 2014.

### Identification

To account for the selection of banks into treatment (government intervention) on their unobservable systemic importance, we employ the two-stage procedure of Heckman (1979). This approach is in line with the previous literature (Berger et al. 2020; Gerhardt and Vander Vennet 2017; Kick et al. 2016). In the first stage, we use a *Probit* model to estimate the probability of a bank receiving policy interventions, as represented by the equation:

1 $$Ln(P_{Intervened\;ij,t}/(1-P_{Intervened\;ij,t}))=\beta_0+\Omega\times{Identifying\;restrictions}_{ij,t-1}+\Phi\times{Bank\;controls}_{ij,t-1}+\Psi\times Market\;\&\;{Macro\;controls}_{j,t-1}+\upsilon_t+\varepsilon_{ij,t}$$

where *P*_*Intervened ij,t*_ represents the probability that bank i receives (one or more) policy interventions in quarter t, *Identifying restrictions*_*ij,t-1*_ is a set of exclusion restrictions that explain the decision of government to provide financial assistance to bank i, and *Bank controls*_*ij,t-1*_ represents differences in risk profiles among banks (size, leverage, credit risk, liquidity and profitability). To account for heterogeneity among different banking systems and economies, we include banking market controls (*Market controls*_*j,t-1*_) and macroeconomic controls (*Macro controls*_*j,t-1*_), which are at the country level. The specifications include year fixed effects* (ʋ*_*t*_*)* to control for unobserved heterogeneity. *ε*_*ij,t*_ is an *iid* error term specific to bank i from country j in quarter t. The explanatory variables are lagged by one period. Variables are winsorized at the 1st and 99th percentiles. The results are corrected for heteroskedasticity and correlation using bank-level clustered standard errors.^6^ The empirical specification is run for the full sample of 83 banks, and the period accounts for 40 quarters during 2005–2014.

The Probit model includes several identifying restrictions that are excluded from the 2^nd^-stage OLS specifications, i.e., political stability, vote share of nongovernment parties, legislative and executive elections dummy, and prevalence. The political stability index measures the stability of political institutions, with higher values indicating greater political stability. The vote share of nongovernment parties reflects the vote share of parties other than the governing parties. The legislative and executive elections dummy is a variable that takes the value 1 if a legislative or executive election took place in the quarter before bailouts were provided. Prevalence is the index developed by Braun and Raddatz (2010) and measures the prevalence of connectedness through the ratio of actual to expected number of political connections (i.e., in essence the number of cases within a country where a former politician later sits on a bank`s board).^7^ The political stability index is from the World Governance Indicators database, the vote share of nongovernment parties and the legislative and executive elections dummy are from the Database of Political Institutions of Cruz et al. (2016), and the prevalence index is from Braun and Raddatz (2010). The choice of the exclusion restrictions follows the literature suggesting that political institutions and electoral cycles influence banks' probability of receiving financial assistance (Brown and Dinc 2005; Liu and Ngo 2014; Behn et al. 2017; Berger et al. 2020).

An important condition that the identifying covariates must satisfy is that they should directly affect the probability of intervention (i.e., one or more of the exclusion variables) but not be correlated with systemic risk. To verify the validity of the exclusion variables, we assess their explanatory power for the likelihood of bailouts and their orthogonality with systemic risk. Following Dam and Koetter (2012), we employ two econometric tests. First, using a weak identification test, i.e., a pseudo Kleibergen‒Paap F test, we check whether the set of identifying restrictions have sufficient explanatory power for the likelihood of intervention in the 1^st^-stage equation. Second, we examine the exogeneity of the excluded covariates, i.e., whether they are uncorrelated with systemic risk, employing a pseudo Hansen J test.^8^

### Baseline model: short-term and long-term effects

In the second stage of the Heckman (1979) procedure, we examine the impact of emergency policy interventions on systemic risk using an *OLS fixed effects* model for the sample restricted to the 30 rescued banks. To control for sample selection bias, the inverse Mills ratio generated by the Probit model in Eq. (1) is included. The following baseline model specification is run for the restricted sample of banks that received bailouts:

2 $${SystemicRisk}_{ij,t}=\beta_0+\beta_1\times{Policy\;interventions}_{ij,\;event\;window}+\beta_2\times{IMR}_{ij,t-1}+\Phi\times{Bank\;controls}_{ij,t-1}+\Psi\times Market\;\&\;{Macro\;controls}_{j,t-1}+\varphi_i+\upsilon_t+\mu_{jt}+\varepsilon_{ij,t}$$

The dependent variable is represented by the contribution of bank i from country j to systemic risk in quarter t. The data represent the values of bank-level systemic risk indicators estimated on a weekly basis using the *MES* methodology proposed by Acharya et al. (2017).^9^ The *MES* assesses the marginal contribution of a bank to the total capital shortfall of the system. Using this methodology, we estimate the average return on bank i’s market capitalization in the weeks in which the total market capitalization of the system experiences the worst 1% of its outcomes (i.e., the expected loss of bank i’s market equity returns conditional on the system’s market equity returns exceeding the *Value at Risk* limits). We start by determining the conditional *Expected Shortfall (ES)* of the system’s returns as follows: ${ES}_{t}^{sys}=$ $E\left[R_{Market\;Equity,t}^{sys}\vert R_{Market\;Equity,t}^{sys}\leq{VaR}_t^{sys}\right],$ where $R_{Market\;Equity,t}^{sys}$ is the return of the system’s market capitalization and ${VaR}_{t}^{sys}$ is the *Value at Risk* indicator that expresses the maximum possible loss that the system can register for a given confidence level α (i.e., 1%) over a specific period of time.^10^ To estimate the values of *MES*, we use a multivariate *GARCH-DCC* specification, a refinement proposed by Brownlees and Engle (2017) that accounts for time-varying volatility and correlation. Online Appendix 4.1 provides a detailed description of the estimations. To merge the quarterly balance sheet and macroeconomic variables, we transform the weekly values of the systemic risk indicators to quarterly frequency by summing them up for each bank within each quarter. We express the systemic risk indicators (losses) as positive numbers; hence, higher values denote greater systemic importance.^11^

The main regressors of interest are represented by the emergency rescue measures taken by the national authorities of country j and implemented by bank i during the event window (*Policy interventions*_*ij, event window*_). These take the form of state guarantees, recapitalizations, and liquidity injections. They are represented by continuous variables obtained by dividing the volume of bailouts by the size of the banks. First, we assess the *short-run* effects of policy interventions by employing an event window starting one quarter before the intervention and ending one quarter after the intervention. Interventions take the same value within this event window (i.e., from t-1 to t + 1). However, for some banks’ rescue packages, a longer period might be required for their effects to be fully realized. Therefore, in the next step, we analyze the *long-run* impact of bailouts on systemic risk by employing an event window approach starting one quarter before the intervention is implemented by bank i and ending the quarter in which the bailout is paid back (i.e., through t-1 to t + n). Under this framework, the volume of bailouts as a share of total assets takes the same value during the event window.^12^ A negative *β*_*1*_ coefficient is associated with a decrease in systemic risk contribution for the rescued banks after they receive the assistance package from government.^13^

*IMR*_*ij,t-1*_ represents the inverse Mills ratio generated by the *Probit model* in Eq. (1). The descriptions of the other regressors are similar to those for Eq. (1). The specifications include bank fixed effects* (φ*_*i*_*)*, year fixed effects* (ʋ*_*t*_*),* and/or country × year fixed effects (*μ*_*ij*_) to control for unobserved heterogeneity and shocks that affected our sample countries, such as regulatory changes.^14^*ε*_*ij,t*_ is an *iid* error term specific to bank i from country j in quarter t. The explanatory variables are lagged by one period. Several alternative models that include other proxies for bank-level risk profiles are estimated to test the robustness of the results. Variables are winsorized at the 1^st^ and 99^th^ percentiles. The results are corrected for heteroskedasticity and correlation using bank-level clustered standard errors,^15^ especially in the context that the dependent variables are preestimated.

A limitation of our analysis is that causality cannot be established with certainty due to the lack of a natural experimental setting within our framework. To address this issue, we provide alternative explanations to the maximum extent possible.

**Fig. 1:**
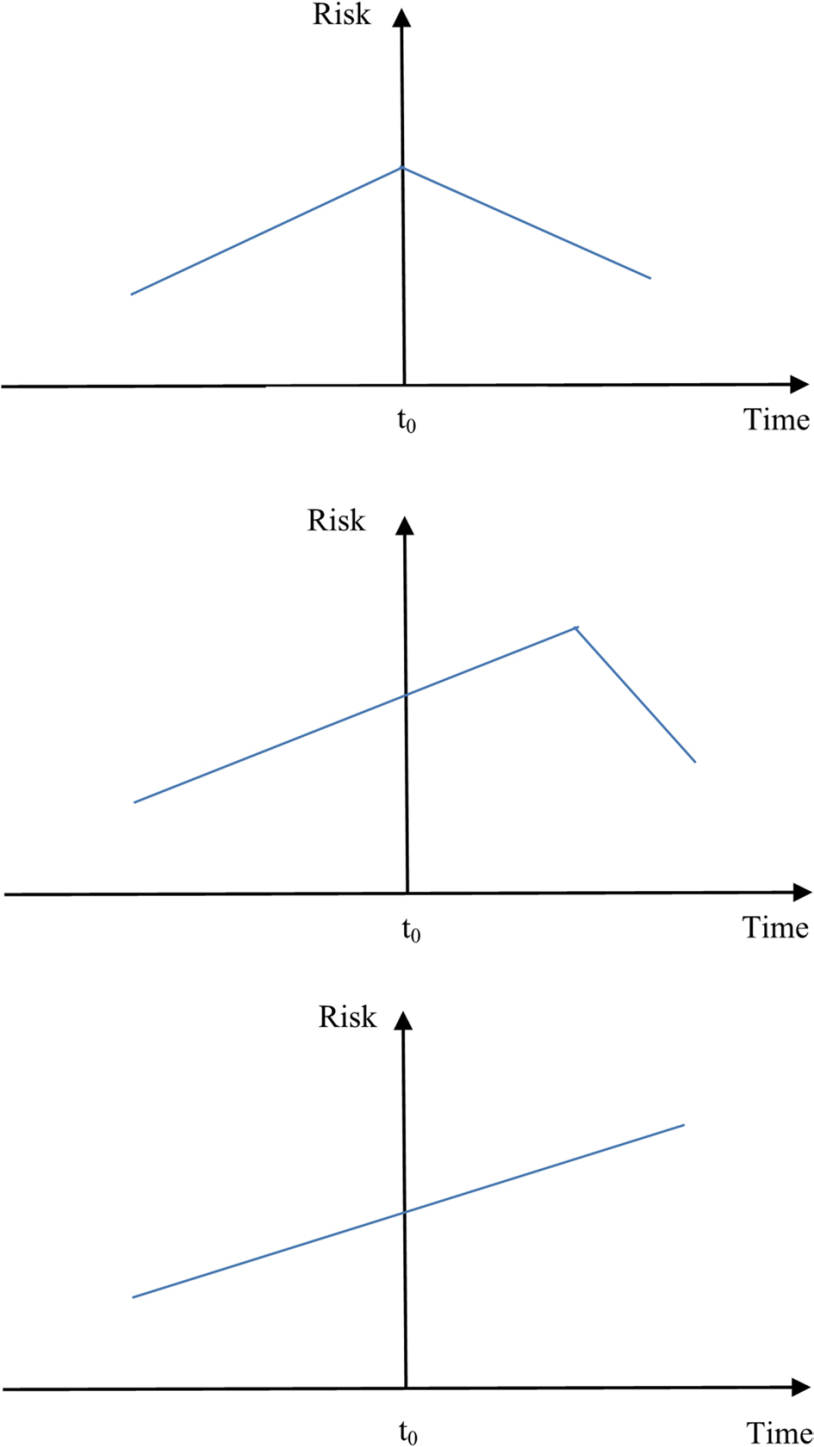
Hypothesis development**.** This figure presents the hypotheses developed for the impact of policy interventions on systemic risk

Policy interventions can exert an immediate effect on systemic risk, a delayed effect, or no effect at all, as depicted in Fig. 1. Moreover, the beginning of our event windows likely precedes the arrival in the market of information about the intervention. Regulators aim to fix systemic distress through financial assistance packages and usually provide them after risk is realized. Thus, declines in the equity value of rescued banks are likely during the time before intervention. An “immediate effect” would imply a prompt drop in systemic risk. However, interventions might not succeed immediately and might lead to a “delayed effect” characterized by an increase in systemic risk first and then a reduction, or measured systemic risk may increase before the intervention and be reversed slowly. As our dependent variable incorporates pre-intervention risk realizations, interventions can be positively linked with systemic risk for a period after their implementation. In addition, European governments faced credibility concerns during the GFC and the ESDC; therefore, declines in equity value may still have occurred after the intervention. Finally, “no effect” might indicate that systemic risk increases before the intervention were fully reversed by the end of the window. The financial health of some banks might not have recovered after the intervention, leading their stock returns to continue to underperform and increasing their contribution to systemic risk even more. Nevertheless, regulators imposed restrictions on the rescued banks and imposed fees during the duration of the bailouts, which could result in even higher systemic risk.^16^

### Further analysis. Transmission channels

#### Restrictions

Policy interventions may have increased measured systemic risk in the long or the short run due to costs associated with regulatory restrictions. In many situations, bonuses were prohibited, and dividends could be distributed only to the government. Additionally, the government occupied seats on some supervisory boards, especially in the case of recapitalizations. In addition, interventions could imply a series of commitments, such as divestments, acquisition bans, or price leadership bans, which can affect investors’ expectations about the future profitability of the bank.^17^ For the banks in our sample, the restrictions were imposed temporarily by the regulator until the intervention was unwound.

To explore the impact of regulatory restrictions on the relation between emergency rescue actions and systemic risk, we consider the following constraints: supervisory board intrusions, management pay limitations, and capital payout bans. Intrusions on supervisory boards may ensure stricter supervision of investment and lending practices, while caps on executive compensation are likely to reduce portfolio risk (Dam and Kotter 2012). Refraining from paying dividends or from buybacks could improve banks’ financial health, as retained earnings increase banks’ capacity to rebuild capital buffers and promote lending. On the other hand, as capital payout limits have strong implications for shareholders, it is likely that they incentivize managers to increase portfolio risk to repay the bailout faster so that the regulator removes the restriction. Acharya and Yorulmazer (2008) show that the government’s stake in the bank should be large enough to overcome risk-taking incentives.

As our MES measure captures both systemic risk realizations and forward-looking systemic risk, the effect of regulatory restrictions on the relation between interventions and systemic risk could be twofold. If shareholders perceive regulatory restrictions to be effective tools for moving portfolio risk toward optimal levels and assuring stricter monitoring of the bank, then the market valuation of more restricted rescued banks is likely to be higher than that of less restricted rescued banks, leading to lower measured systemic importance for the former. In turn, if investors perceive the regulatory burden to be costly, then stricter restrictions may lead to underperforming stock returns and therefore higher measured systemic risk for more restricted than for less restricted bailed-out banks. This translates into a higher systemic importance for bailed-out banks when regulators impose tighter restrictions.

The impact of restrictions on the link between policy interventions and systemic risk is examined with the following specification:

3 $${SystemicRisk}_{ij,t}=\beta_0+\beta_1\times{Policy\;interventions}_{ij,\;event\;window}+\beta_2\times{Policy\;interventions}_{ij,\;event\;window}\times{Restrictions}_{ij,t-1}+\beta_3\times{Restrictions}_{ij,t-1}+\beta_4\times{IMR}_{ij,t-1}+\Phi\times Bank\;{controls}_{ij,t-1}+\Psi\times Market\;\&\;{Macro\;controls}_{j,t-1}+\mu_{jt}+\varepsilon_{ij,t}$$

In addition to Eq. (2), we include the interaction term of policy interventions with the restrictions imposed by the regulator. The latter are captured by dummy variables that reflect the following dimensions: supervisory board intrusions, management pay limitations, and capital payout bans. The coefficient *β*_*2*_ should be negative and significant if such restrictions reduce the systemic importance of banks when interventions are implemented and positive otherwise. As in the baseline specification, we use the same bank-level, market and macro controls. Additionally, we account for sample selection bias by including the inverse Mills ratio generated by the *Probit model* in Eq. (1). The strategy involves estimating the empirical models separately for each interaction of policy interventions with the restrictions using *OLS FE* for the restricted sample of rescued banks.

#### Bank risk

The interventions made by supervisory authorities may have different impacts on systemic risk across banks with varying risk profiles. While in the short run a bank’s portfolio cannot change swiftly, in the long run, banks can adjust their portfolio and be more inclined to manifest moral hazard behavior. The mechanism by which increased moral hazard affects systemic risk is usually a change in the bank’s risk strategy. To further explore the effects of interventions on systemic risk, we examine the impact of banks’ risk strategies on the relation between emergency rescue actions and systemic risk, focusing on the following channels: size, leverage, and profitability.

##### Size

As governments are more likely to bail out TBTF institutions, rescued banks may speculate on this behavior and increase their size to benefit from future interventions. This strategy can increase their fragility, and their contribution to systemic risk may be intensified in comparison with that of rescued banks of a smaller size. A number of empirical papers find that rescue packages provided to large banks stimulate them to focus on riskier activities and complex projects that are difficult to manage (Duchin and Sosyura 2014; Laeven et al. 2016), invest in illiquid assets (Cao and Illing 2008), or take on excessive credit risk (Gropp et al. 2014). On the other hand, bailing out a bank and confirming its TBTF status might cause the bank to downsize after the intervention due to more certainty about its TBTF status. In this case, the realization of distress, and therefore the systemic importance, may be reduced for larger rescued banks in comparison to that of smaller rescued banks.

##### Leverage

The policy interventions implemented in the European banking sector were provided to viable financial institutions, which had to meet certain capital ratios.^18^ Choi (2014) theoretically shows that recapitalizing safer institutions is effective in reducing contagion spillovers. By increasing the charter value, these banks may opt for safer investments, and their appetite for risk-taking may be reduced (Cordella and Yeyati 2003). In this way, we expect measured systemic risk to be diminished for rescued banks with a higher capitalization (i.e., above the sample median), as these banks have a greater loss-absorbing capacity than rescued banks with a lower capital ratio. At the same time, empirical evidence suggests that banks with higher excess regulatory capital may opt to take risky projects to generate returns for shareholders (Huang and Ratnovski 2009; Perotti et al. 2011). This may be especially likely to take place for banks injected with capital, as shareholders’ stakes are usually diluted by the intervention. Thus, bailed-out banks with a greater capitalization may also have an enhanced contribution to systemic risk in comparison to that of rescued banks with lower capitalization.

##### Profitability

The influence of moral hazard on systemic risk can be exerted through the profitability channel. On the one hand, a bank’s higher profitability can soften the regulatory burden associated with bailouts, which leads to smaller declines in market valuation and therefore a lower systemic risk contribution. On the other hand, more profit may create conditions for banks to borrow more and take on more risk. Martynova et al. (2020), for example, show that profitable banks are more likely to make market-based investments than less profitable banks. This can lead to greater systemic risk for more profitable banks.

To assess these hypotheses, the following regression model extends the baseline specification regarding the impact of interventions on systemic risk:

4 $${SystemicRisk}_{ij,t}=\beta_0+\beta_1\times{Policy\;interventions}_{ij,\;event\;window}+\beta_2\times{Policy\;interventions}_{ij,\;event\;window}\times{Bank\;risk}_{ij,t-1}+\beta_3\times{Bank\;risk}_{ij,t-1}+\beta_4\times{IMR}_{ij,t-1}+\Phi\times{Bank\;controls}_{ij,t-1}+\Psi\times{Market\;\&\;Macro\;controls}_{j,t-1}+\mu_{jt}+\varepsilon_{ij,t}$$

In addition to Eq. (2), we include the interaction term of policy interventions with the bank-level risk strategy indicators. These are dummy variables that reflect the size, leverage, and profitability of the bank at t-1 (i.e., if a bank’s risk is above the median risk of the sample of rescued banks one quarter before intervention).^19^ The coefficient *β*_*2*_ should be positive and significant if risk strategies enhance the systemic importance of banks when they receive bailouts and negative otherwise. The remaining coefficients are as in Eq. (2), and the empirical strategy mimics that of the previous section. We estimate the empirical models separately for each interaction of policy interventions with the risk profile indicators.

## Data

This section presents the sample and the data used for estimating systemic risk measures and the variables employed in the panel regression specifications.

### Sample construction

Our sample consists of 83 publicly listed European banks whose assets totaled more than 20 trillion euros at the end of 2014. They are internationally active and represent 22 European states. The interest in this portfolio is motivated by regulatory considerations, as the group includes large banks identified as G-SIBs (Global Systemically Important Banks) by Financial Supervisory Board but also small local banks that present systemic importance. Among them, 53% are included in the EBA’s stress testing exercise, while 39% are included in the ECB’s Single Supervisory Mechanism framework (Online Appendix 1). We focus only on banks because they are the most important financial intermediaries in Europe. The size variation is considerable within the sample, as total assets at the end of 2014 ranged from approximately 1 billion euros to approximately 2 trillion euros. The average coverage of total banking system assets of the analyzed countries is approximately 49%. We use consolidated statements to capture all cross-border business transactions of international banks.

We start with a sample of 351 active and publicly listed financial institutions from the EU28 area that are included in the Thomson Reuters Financial Datastream within the sector “Banks”. We consider publicly listed banks because the methodology that we use for the estimation of systemic risk indicators is based on market data, which restricts the sample to banks listed on a stock exchange. Furthermore, due to methodology constraints imposed by the systemic risk estimation, we apply several exclusion criteria. First, we exclude banks that do not have weekly market capitalization data available in Datastream for the whole period. Second, we eliminate banks with negative equity.^20^ Third, we include banks that have more than 75% of observations for their quarterly balance sheet data available in Worldscope (Table 1). Finally, to have a balanced sample in terms of size and across countries, we eliminate banks with total assets below 1 billion euros at the end of 2014. These filters lead us to our final sample of 83 banks, out of which 30 institutions received public interventions (Table 2).^21^

**Table 1:** Description of variables. Y represents yearly frequency, Q is quarterly frequency, and, W is weekly frequency. Own C^a^ represents own calculations using data from Worldscope and Datastream, while Own C^b^ are calculations based on data from banks’ financial statements, websites and State Aid Register of European Commission. ECB stands for European Central Bank, GFDB for Global Financial Development Database, SBRS for World Bank Survey of Bank Regulation and Supervision (2003, 2007 and 2011), WDI for World Development Indicators, WGI for World Governance Indicators, DPI for the Database of Political Institutions of Cruz et al. (2016), and BR for Braun and Raddatz (2010)

Variable name	Description and calculation	Frequency	Source
Dependent variables (bank level)			
MES	Marginal expected shortfall expressed in units of percentage loss of the bank’s market equity within a quarter. MES is defined as in Acharya et al. (2017), i.e., the average return on bank’s market capitalization on the weeks the total market capitalization of the sample experienced its 1% worst outcomes. The measure is determined using *DCC—GJR GARCH* method. System is defined by the Market capitalization of the sample. The weekly values are summed up within a quarter. The indicator (loss) is expressed as a positive number, hence higher values denote greater systemic importance	Q	Own C^a^
Delta CoVaR	Contribution to systemic risk expressed in units of percentage loss of the system’s market value of equity within a quarter. Delta CoVaR is defined as in Adrian and Brunnermeier (2016), i.e., the difference of the Value-at-Risk of the system’s market equity conditional on the distress of a particular bank (1% worst outcomes) and the Value-at-Risk of the system’s market equity conditional on the median state of the bank. The measure is determined using *Quantile Regression*, based on weekly market capitalization and a set of domestic and global market indices. The system is the Market capitalization of the sample. The weekly values are summed up within a quarter. The indicator (loss) is expressed as a positive number, higher values denoting greater systemic importance	Q	Own C^a^
Data used for estimating systemic risk			
*Balance sheet data (bank level)*			
Market equity	Market capitalization (bil eur)	W	Datastream
Returns on bank i’s market equity in week t	$\mathrm R_{\mathrm{Market}\;\mathrm{Equity}\;(\mathrm t)}^{\mathrm i}=\frac{\mathrm{Market}\;\mathrm{Equity}_{\mathrm t}^{\mathrm i}}{\mathrm{Market}\;\mathrm{Equity}_{\mathrm t-1}^{\mathrm i}}-1\;\%$	W	Worldscope
Returns on system’s market equity in week t	$\mathrm R_{\mathrm{Market}\;\mathrm{Equity}\;(\mathrm t)}^{\mathrm{sys}}=\sum_{\mathrm i}\frac{\mathrm{Market}\;\mathrm{Equity}_{\mathrm t}^{\mathrm i}}{\sum_{\mathrm i}\mathrm{Market}\;\mathrm{Equity}_{\mathrm t}^{\mathrm i}}\times\mathrm R_{\mathrm{Market}\;\mathrm{Equity},\mathrm t}^{\mathrm i},$ i takes values from 1 to the sample’s number of banks (%)	W	Worldscope
*Financial market indices*			
Government bonds yield	Change in the Euro AAA government bonds yield curve instantaneous forward rate ten-years against one-month residual maturity	W	ECB
Funding liquidity spread	Difference between the Euribor three-month interbank rate and the Euro area government bonds three-month yield curve	W	ECB
Real estate price index	Change in the Real estate price index for Europe	W	Datastream
VSTOXX	Change in the implied volatility index within the Eurozone	W	Bloomberg
Data used for panel regressions			
*Policy interventions (bank level)*			
State guarantees	Guarantees provided by state j to bank i in quarter t (as % of Total assets). On short run they are maintained at the same level from one quarter before the event to one quarter after the event (t-1; t + 1). On long run they are maintained at the same level from one quarter before the event to the quarter they are paid back (t-1; t + n)	Q	Own C^b^
Recapitalizations	Capital injections provided by state j to bank i in quarter t (as % of Total assets). On short run they are maintained at the same level from one quarter before the event to one quarter after the event (t-1; t + 1). On long run they are maintained at the same level from one quarter before the event to the quarter they are paid back (t-1; t + n)	Q	Own C^b^
Liquidity injections	Liquidity injections provided by state j to bank i in quarter t (as % of Total assets). On short run they are maintained at the same level from one quarter before the event to one quarter after the event (t-1; t + 1). On long run they are maintained at the same level from one quarter before the event to the quarter they are paid back (t-1; t + n)	Q	Own C^b^
*Regulatory restrictions (bank level)*			
Supervisory board intrusions	Dummy variable that takes the value 1 if the government appointed members on the supervisory board and 0 otherwise	Q	Own C^b^
Management pay limitations	Dummy variable that takes the value 1 if the government limited the salaries and bonuses of bank’s executives and 0 otherwise	Q	Own C^b^
Capital payout bans	Dummy variable that takes the value 1 if the government prohibited the dividend and other capital payouts and 0 otherwise	Q	Own C^b^
*Bank characteristics (bank level)*			
Beta	Covariance of bank i’s stock returns with the market’s stock returns divided by the variance of the market’s stock returns	Q	Own C^b^
Size	log(Total Assets)	Q	Worldscope
Asset growth	Asset growth relative to average bank asset growth in the bank’s country	Q	Own C^a^
Leverage	Common Equity/Total Assets (%)	Q	Worldscope
Credit risk ratio	Provisions for Loan Losses/Gross Loans (%)	Q	Worldscope
Liquidity ratio	Liquid Assets/Deposits and Short-term Funding (%)	Q	Worldscope
Rollover risk ratio	Deposits and Short-term Funding/Total Deposits and Borrowings (%)	Q	Worldscope
Return on Average Assets (ROAA)	Net Profit/Average Assets (%)	Q	Worldscope
Gross loans share	Gross Loans/Total Assets (%)	Q	Worldscope
Net non-interest margin	Net Non-Interest Income/Gross Revenues (%)	Q	Worldscope
Dummy size	Dummy variable that takes the value 1 if the size of the bank is above the median size of the intervened banks sample	Q	Own C^a^
Dummy leverage	Dummy variable that takes the value 1 if the leverage of the bank is above the median Common Equity to Total Assets ratio of the intervened banks sample	Q	Own C^a^
Dummy profitability	Dummy variable that takes the value 1 if the ROAA ratio of the bank is above the median Net Profit to Average Assets ratio of the intervened banks sample	Q	Own C^a^
*Market & Macro controls (country level)*			
Competition	Boone indicator, a measure of competition in the banking market calculated as the elasticity of profits to marginal costs. The lower the Boone indicator is, the higher the level of competition	A	GFDB
Capital regulatory index	A composite index that measures the amount of regulatory capital banks must hold and the stringency of regulations on the quality capital. The index takes values from 0 (relaxed regulations) to 10 (tight regulations)	A	SBRS
Sovereign debt	Sovereign debt holdings of the banking system as share in GDP	A	ECB
Inflation	Inflation measured by the consumer price index, reflecting the annual percentage change in the cost to the average consumer of acquiring a basket of goods and services that may be fixed or changed at specified intervals	A	WDI
GDP growth	Gross domestic product at market prices, calculated as % change on previous period, based on 2005 = 100	A	WDI
Crisis	Dummy variable that takes the value 1 after the Lehman Brothers collapse and 0 otherwise	0/1	
GFC crisis	Dummy variable that takes the value 1 during 2008 q3 – 2009 q4 and 0 otherwise	0/1	
SVG crisis	Dummy variable that takes the value 1 during 2010 q1 – 2012 q4 and 0 otherwise	0/1	
*Exclusion restrictions (country level)*			
Political stability index	A composite index that measures the stability of the political institutions. The index takes values from -2.5 (weak) to 2.5 (strong)	A	WGI
Vote share non-government parties	The vote share of parties other than the government ones	A	DPI
Legislative & executive elections	Dummy variable that takes the value 1 if a legislative or executive election took place in the quarter when bailouts were provided	Q	DPI
Prevalence	An index that measures the prevalence of connectedness through the ratio of actual to expected number of political connections (i.e., number of cases within a country with a former politician who later sits on board). The probability that a former politician later sits on a bank’s board is derived by assuming that the connections are selected randomly with replacement from a common pool. This probability gives the expected number of political connections within a country	-	BR

**Table 2:** The distribution of banks. The calculations are based on Worldscope data for Total assets of our sample and European Banking Federation data for Total assets of the banking system in each country at year end 2014

Country	Number of banks	Total assets sample (billion €)	Total assets country (billion €)	Total assets sample / Total assets banking system (%)
Austria	6	354.41	915.11	38.73%
Belgium	1	245.17	1021.57	24.00%
Bulgaria	1	4.52	47.41	9.54%
Cyprus	2	34.34	90.20	38.07%
Czech Republic	1	34.39	190.87	18.02%
Denmark	8	576.43	1048.30	54.99%
Finland	1	4.29	525.31	0.82%
France	7	5833.12	7881.63	74.01%
Germany	4	2283.04	7528.95	30.32%
Hungary	1	34.87	116.06	30.05%
Ireland	2	237.26	1016.95	23.33%
Italy	12	1876.55	4047.89	46.36%
Lithuania	1	1.64	24.04	6.82%
Malta	3	16.66	50.33	33.10%
Netherlands	2	1010.12	2250.13	44.89%
Poland	10	221.08	361.63	61.13%
Portugal	3	118.99	515.33	23.09%
Romania	1	8.00	91.40	8.75%
Slovakia	4	24.76	61.13	40.50%
Spain	6	2619.00	3150.74	83.12%
Sweden	4	1476.29	1514.50	97.48%
United Kingdom	3	3047.90	8895.35	34.26%
Total	83	20,062.81	41,344.83	48.53%
EU-28			42,520.53	47.18%

### Systemic risk variables

The data required for our systemic risk estimations span 2005 to 2014. We chose this period because it allows us to track the evolution of systemic risk during two financial crisis, i.e. the 2008 global financial crisis and the 2010 European sovereign debt crisis. Following Brunnermeier and Oehmke (2013), we estimate systemic risk during the whole period to account for the buildup phase in the precrisis and the propagation phase during the crisis. The impact of policy interventions on systemic risk is analyzed during the same time span. Although there have been important regulatory changes after 2014, most of the bailout packages were provided to European banks during 2008–2012, according to the State Aid Register data of European Commission.

**Fig. 2:**
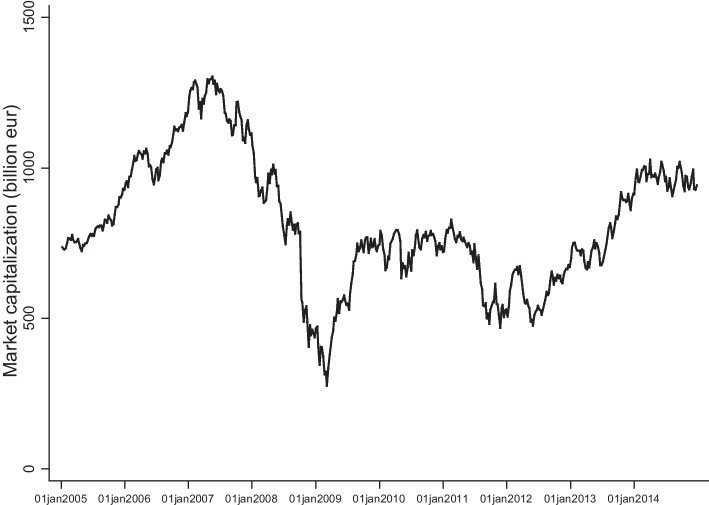
Evolution of the sample’s market capitalization This figure presents the evolution of the market capitalization for 83 European banks from 2005 to 2014. The values are expressed in billion euros

The systemic risk measures are estimated separately for each bank using weekly returns extracted from Datastream (see Table 1 and Online Appendix 4 for computation details). Figure 2 presents the evolution of our sample’s market capitalization. This corresponds to the 83 banks analyzed over 521 weeks (2005–2014). Due to the deteriorating economic conditions in international financial markets, market equity shows a downturn in the first phase of the crisis (2008–2009), decreasing by more than 70% in comparison with its maximum value, reached in the middle of 2007. There were signs of recovery during 2009–2010, but the market equity started declining again at the end of 2011 when the European sovereign debt crisis took off.

The summary statistics of the systemic risk indicators are reported in Table 3 (Panel A). The data corresponding to the *Marginal Expected Shortfall* model reveal that for 2005–2014, the quarterly average contribution to systemic risk of all banks translates to an approximately 69% loss of the banks’ market capitalization. The statistics resulting from the *Conditional Value at Risk* measure, which we employ as a robustness check, show that banks’ marginal contribution to systemic risk represents an approximately 39% loss of the system’s market capitalization within a quarter.

**Table 3:** Summary statistics of systemic risk indicators. The table reports the summary statistics of the dependent variables during 2005–2014. Panel A reports the output for the whole sample. Panel B provides the difference in means analysis between non-intervened banks and banks affected by policy interventions, based on a two-sample t-test with unequal variance. Definition of variables is provided in Table 1. Values are expressed in units of percentage loss of the banks’ market equity within a quarter (MES), and, units of percentage loss of the system’s market equity within a quarter (Delta CoVaR)

Panel A. All sample																									
Variables	Unit		Mean			Std. dev.			Min			p25			p50				p75			Max		No. obs.	
MES	Quarterly % loss of banks’ Market equity		69.30			43.01			0.00			33.75			67.76				100.11			149.09		3320	
Delta CoVaR	Quarterly % loss of system’s Market equity		38.74			25.26			0.00			19.32			37.69				55.06			92.12		3320	
Panel B. Intervened versus non-intervened banks																									
	Non-intervened bank											Intervened banks										Difference in means (Intervened vs. Non-intervened)			
Variables	Unit	Mean		Std. dev.	Min		p25	p50	p75	Max	No. obs.	Mean	Std. dev.	Min		p25	p50	p75		Max	No. obs.				
*State guarantees*																									
MES	%	62.32		41.85	0.00		29.00	59.14	89.79	149.09	2560	92.83	38.26	0.00		67.87	94.36	120.28		149.09	760		30.51		^***^
Delta CoVaR	%	37.16		25.01	0.00		16.69	37.20	52.91	92.12	2560	44.06	25.40	0.00		24.04	42.07	62.31		92.12	760		6.90		^***^
*Recapitalizations*																									
MES	%	61.68		41.52	0.00		28.31	58.19	90.83	149.09	2440	90.44	39.91	0.00		65.32	88.29	121.38		149.09	880		28.76		^***^
Delta CoVaR	%	36.96		25.14	0.00		16.22	36.39	53.47	92.12	2440	43.67	24.96	0.00		26.46	41.37	59.37		92.12	880		6.71		^***^
*Liquidity injections*																									
MES	%	63.02		41.60	0.00		29.45	60.23	91.34	149.09	2720	97.78	37.46	0.00		71.70	99.62	129.76		149.09	600		34.76		^***^
Delta CoVaR	%	37.12		24.86	0.00		17.15	36.58	52.99	92.12	2720	46.12	25.76	0.00		25.62	44.68	62.37		92.12	600		9.00		^***^

The main features are compared between unrescued banks and banks affected by rescue packages (Table 3 Panel B). Overall, the difference in means analysis shows that the mean contribution to systemic risk for the whole sample period is larger for banks that received state guarantees, capital injections or liquidity injections than for nonaffected banks.^22^

**Fig. 3:**
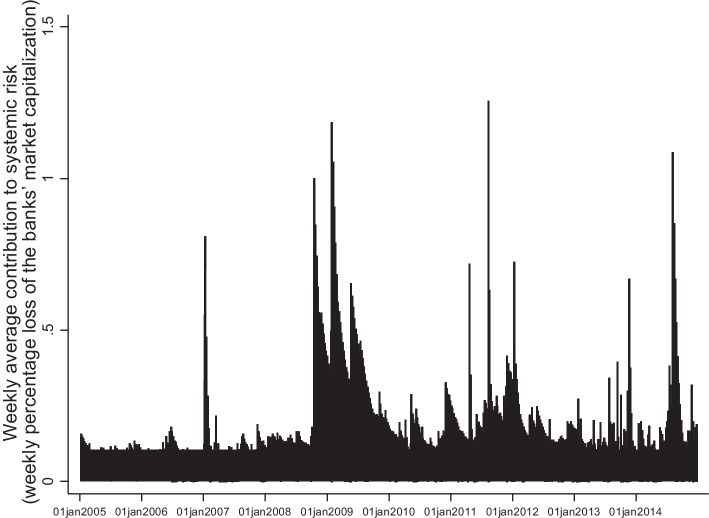
Weekly contribution to systemic risk of banks. This figure presents the weekly evolution of systemic risk for all 83 banks in our sample during 2005–2014. Values obtained for each bank are averaged on a weekly base for the whole sample. The output corresponds to the banks’ contribution to systemic risk determined via the Marginal Expected Shortfall model. We express the systemic risk indicators as positive numbers (weekly percentage loss of the banks’ market capitalization), hence higher values denote greater systemic importance

Figure 3 presents the weekly average contribution to systemic risk of all banks in our sample during 2005–2014. The graph reveals an increase in risk during the Lehman collapse in September 2008 and the European sovereign debt crisis. Our systemic risk measure is very likely to reflect both risk realization and forward-looking risk. Even though the governments of European member states intervened promptly with financial assistance programs, interventions were usually applied after the realization of risk. Therefore, declines in the market equity of bailed-out banks were likely during the period before intervention and may have persisted for a period after. Similarly to us, Black et al. (2016) find that the systemic risk of the European banking system increased during the crisis, reaching a peak during the sovereign debt crisis in Europe.

### Emergency policy interventions

The impact on systemic risk of the emergency measures taken by European member states during the global financial crisis and the European sovereign debt crisis is analyzed for each of several types of policy interventions. To limit the negative spillovers in the banking system and to ensure financial stability, supervisory authorities used a broad range of mechanisms which we group into three categories (1) state guarantees, (2) recapitalizations (capital injections), and (3) liquidity injections. These are described in Online Appendix 2, which provides details on the type, size, and time of implementation of the interventions. Banks from our sample received public guarantees for bond issues, senior notes or other forms of debt; recapitalizations in the form of hybrid capital, participation capital, preferred shares, deeply subordinated perpetual notes or contingent convertible subordinated bonds (CoCos); and liquidity injections consisting of loan facilities, swap facilities, illiquid asset back-up facilities or asset protection schemes (APSs). We hand-collect the dataset from banks’ annual reports, financial statements, websites and the State Aid Register of the European Commission.

All three types of bailouts were applied to the banks in our sample during 2008–2014 (Online Appendix 3). In terms of value, liquidity injections lead with an average size of approximately 4% of banks’ total assets, followed by state guarantees (3% of total assets) and recapitalizations (2% of total assets). The aim of guarantee schemes is to ensure the supply of liquidity in the interbank market or to prevent bank runs. Recapitalizations are intended to strengthen the capital base of banks. Liquidity injections are given to limit the probability of runs and to encourage bank participation in asset markets, thereby limiting financial instability. They are expected to generate higher liquidity and greater transparency. The guarantees and recapitalizations were more country-wide in nature, as the schemes were aimed at restoring confidence, while the liquidity measures targeted the exposure to losses by individual banks (Panetta et al. 2009).^23^

Table 4 provides descriptive statistics of the financial support provided by the government for the entire sample in Panel A and for the sample restricted to banks with intervention events in Panel B. In sum, our sample was exposed to 106 policy intervention events: 36 events corresponding to state guarantees, 35 events related to recapitalizations, and 35 events linked with liquidity injections (among which 8 are related to APSs and 27 to other types of emergency liquidity schemes). Out of 83 banks, 30 implemented these types of policies: 6 banks received all three types of interventions, 14 banks applied two types of interventions, and 10 banks relied on a single intervention measure. The rescued banks represent 15 countries (out of the 22 included in our sample), among which in seven countries more than two banks were affected. From the 30 rescued banks in our sample, 18 were released from the bailouts before 2014.

**Table 4:** Descriptive statistics of explanatory variables. The definition of variables is provided in Table 1. Statistics are based on data spanning from 2005 to 2014. Panel A shows statistics for the full sample of 83 banks. Panel B depicts statistics for the sample restricted to the intervention events

Variables	Unit	Mean	Std. dev.	p25	p50	p75	Min	Max	No. obs.
Policy interventions (bank level)									
A. Full sample									
State guarantees (% of Total assets)	%	0.07	1.67	0.00	0.00	0.00	0.00	7.34	3320
Recapitalizations (% of Total assets)	%	0.03	0.79	0.00	0.00	0.00	0.00	11.67	3320
Liquidity injections (% of Total assets)	%	0.05	1.03	0.00	0.00	0.00	0.00	40.46	3320
Supervisory board intrusions	0/1	0.12	0.33	0.00	0.00	0.00	0.00	1.00	3320
Management pay limitations	0/1	0.24	0.43	0.00	0.00	0.00	0.00	1.00	3320
Capital payout bans	0/1	0.19	0.39	0.00	0.00	0.00	0.00	1.00	3320
B. Intervention events sample									
State guarantees (% of Total assets)	%	2.67	1.77	1.51	1.98	3.44	0.05	7.34	36
Recapitalizations (% of Total assets)	%	1.60	2.10	0.50	1.02	1.95	0.11	11.67	35
Liquidity injections (% of Total assets)	%	4.41	9.13	0.16	0.50	4.30	0.09	40.46	35
Supervisory board intrusions	0/1	0.28	0.45	0.00	0.00	0.00	1.00	1.00	85
Management pay limitations	0/1	0.67	0.47	0.00	0.00	1.00	1.00	1.00	85
Capital payout bans	0/1	0.41	0.50	0.00	0.00	0.00	1.00	1.00	85
Risk profile indicators (bank level)									
Beta	–	−0.04	0.35	−0.16	−0.03	0.07	−4.99	2.80	3283
Size	Log(bil. €)	3.74	2.16	2.14	3.62	5.50	−0.46	7.60	2381
Asset growth	%	0.43	3.80	−0.21	0.21	0.85	−18.03	21.95	2371
Leverage	%	7.40	3.27	4.91	6.88	9.13	1.08	24.50	2381
Credit risk ratio	%	0.89	1.02	0.32	0.65	1.17	−1.57	13.87	2361
Liquidity ratio	%	32.43	28.97	13.61	23.65	40.44	2.31	223.38	2379
Rollover risk ratio	%	77.17	17.53	63.87	78.83	93.48	14.15	100.00	2366
ROAA	%	0.62	0.95	0.24	0.55	0.93	−10.46	10.64	2394
Gross loans share	%	61.12	16.20	53.95	63.70	72.99	3.34	90.39	2378
Net non-interest margin	%	−22.91	35.11	−31.29	−20.47	−10.22	−859.52	808.46	2360
Market & Macro conditions (country level)									
Competition (Boone indicator)	–	−0.05	0.06	−0.09	−0.04	−0.03	−0.20	0.22	2988
Capital regulatory index	–	5.98	2.03	4.00	6.00	8.00	3.00	11.00	3320
Sovereign debt	–	68.00	29.59	44.27	64.52	86.06	11.95	135.37	3320
Inflation	%	2.11	1.47	1.22	2.06	2.81	−4.48	12.35	3320
GDP growth	%	0.94	3.06	−0.63	1.30	2.55	−13.86	12.41	3304
Exclusion restrictions (country level)									
Political stability index	–	0.74	0.39	0.50	0.84	1.04	−0.47	1.59	3320
Vote share of non-government parties	%	0.94	3.00	0.00	0.00	0.00	0.00	23.40	3240
Legislative & executive elections	0/1	0.09	0.30	0.00	0.00	0.00	0.00	2.00	3320
Polarization	–	1.25	0.91	0.00	2.00	2.00	0.00	2.00	3008

In most cases, bailouts come at a cost for banks. For example, banks often paid a fee, usually linked to the risk of the financial institution (i.e., its rating), executive compensation is limited, and dividends could be distributed only to the government (Petrovic and Tutsch 2009). We account for these features by considering a set of restrictions imposed by regulators during the duration of bailouts: supervisory board intrusions, management pay limitations, and capital payout bans. They are expressed as dummy variables, and their definitions are provided in Table 1. Among the policy intervention events from our sample, 28% are associated with supervisory board intrusions, 67% have limitations on executive compensation, and 41% are linked with capital payout restrictions.^24^

### Bank-level controls

To account for different risk strategies, we control for the risk profiles of banks one quarter before intervention. Prior studies suggest that bank characteristics such as size, leverage, profitability, and credit and liquidity risk were key drivers of systemic risk during the most recent financial crisis (Demirgüç-Kunt and Huizinga 2010; Tarashev et al. 2010; Acharya et al. 2012; Mayordomo et al. 2014). In line with the literature, the following risk profile indicators are used: (1) size (logarithm of total assets); (2) asset growth (asset growth relative to average bank asset growth in the bank’s country); (3) leverage (common equity to total assets ratio and Tier 1 regulatory capital ratio in robustness exercises); (4) the quality of the loan portfolio (provisions for loan losses to gross loans); (5) liquidity ratio (liquid assets to deposits and short-term funding); (6) rollover risk ratio (deposits and short-term funding to total deposits and borrowings); and (7) profitability represented by the ROAA ratio (net profit to average assets). Our presumption is that larger size, asset growth, credit risk, and rollover risk are associated with a higher level of systemic risk, while higher capitalization, liquidity, and profitability are associated with a lower level. Additionally, we capture the orientation of banks’ business toward traditional and nontraditional activities by including the share of lending activity (gross loans to total assets) and the net noninterest margin (net noninterest income to gross revenues). Previous studies show that systemic risk is associated with a high share of nontraditional activity (Brunnermeier et al. 2020; Demirgüç-Kunt and Huizinga 2010). Additionally, we capture the comovement of bank value with financial system value by including the beta coefficient, computed as the covariance of bank i’s stock returns with the market’s stock returns divided by the variance of the market’s stock returns. The variables are extracted from Worldscope, and their definitions are given in Table 1.^25^ The descriptive statistics presented in Table 4 show that on average during 2005–2014, banks from our sample had a Tier 1 ratio of 11%, liquidity ratio of 32%, credit risk ratio of 1% and gross loans shares of 61%. These statistics suggest that on average, the institutions are well capitalized and have a good liquidity situation and a high-quality loan portfolio. Additionally, they are more oriented toward traditional banking business.

### Macro controls

Following previous studies (Girardi and Ergün, 2013; Anginer et al. 2014a; Weiß et al. 2014a, b), we control for the banking market and macroeconomic environment. Accounting for the particularities of each national banking sector, we consider the intensity of competition − or lack thereof − expressed by the Boone indicator,^26^ which has been found to be associated with a reduced contribution to systemic risk (Anginer et al. 2014a). Next, we account for the strictness of prudential regulations regarding initial and overall capital held by banks. As a proxy, we use the capital regulatory index provided by the Bank Regulation and Supervision Database of the World Bank and calculated as in Barth et al. (2013).^27^ Additionally, we employ the sovereign debt holdings of the banking system as a share in GDP and the inflation rate and GDP growth as macro controls. We expect systemic risk to increase with the deterioration of macro conditions. Finally, we include in our specifications a dummy variable that reflects the global financial crisis *(Dummy GFC)* and a dummy variable that accounts for the European sovereign debt crisis *(Dummy SVG)* to control for the dynamics of these crises that affected the evolution of systemic risk. The variables are extracted from the World Development Indicators, Global Financial Development and European Central Bank databases. Their definitions are given in Table 1, and the descriptive statistics are given in Table 4.

## Empirical results

This section presents the results of the regressions with the systemic risk measures as our dependent variables and policy interventions as the main determinants. First, we discuss the influence of policy interventions on banks’ systemic importance. Both short- and long-term effects are assessed. Second, we examine how regulatory restrictions and banks’ risk strategies affect the impact of emergency rescue actions on systemic risk. The main regressors of interest include the volume of emergency rescue packages received by bank i from government j during the event window as a share of banks’ total assets. In the case of short-run estimations, these bailouts take the same value from one quarter before intervention to one quarter after. In the case of long-run estimations, the bailouts take the same value from one quarter before intervention to the quarter in which they are paid back.

### Emergency policy interventions and systemic risk: Baseline results

The output for the 1^st^-stage Heckman (1979) selection model is presented in Table 5 Panel A. The results reveal that greater political stability significantly increases the probability of the government providing bailouts to financial institutions. The vote share of nongovernment parties enters the model significantly, with a positive sign, suggesting that in countries with higher political competition where the vote share of parties other than the government parties is higher, the regulator is more likely to implement policy interventions. Prevalence is significant as well. The F statistic associated with the pseudo Kleibergen‒Paap F test indicates that the set of identifying covariates has sufficient predictive power to explain the likelihood of the government providing financial assistance.^28^ Additionally, the p value corresponding to the pseudo Hansen J test validates the orthogonality between the exclusion restrictions and systemic risk. Therefore, our set of instruments contains additional information on the likelihood of bailouts and meets the validity conditions.^29^

#### Short-term effects

Table 5 Panel B shows the estimation results for the *OLS fixed effects* regression presented in Eq. (2). The dependent variable captures the banks’ contribution to systemic risk, estimated using the *Marginal Expected Shortfall* methodology. Model (2) provides a baseline specification that includes all policy interventions, bank characteristics, micro and macro controls, bank fixed effects and year fixed effects.^30^ Model (3) includes additional country × year fixed effects, and Model (4) includes bank fixed effects and country × year fixed effects.

We consider Column (4), which includes all bank and macro characteristics, as well as bank fixed effects and country × year fixed effects, our benchmark specification. In what follows, the interpretation of the empirical results is detailed for this model.^31^ A negative coefficient is related to a lower systemic importance, while a positive coefficient is linked to an increased contribution of banks to systemic risk.^32^

Overall, the results specific to the short run validate the “delayed effects” hypothesis and indicate that interventions do not succeed immediately. There are several possible explanations for our findings. First, as governments usually provide bailouts after risk is realized, declines in the market equity of bailed-out banks are likely before intervention and may persist for a period thereafter. This leads to a higher measured systemic risk contribution, considering the nature of our MES variable, which captures risk realizations as well as forward-looking risk. Second, such delayed success might have arisen due to some European states’ lack of credibility in providing financial assistance during the GFC and the ESDC. Moreover, the imposition of regulatory constraints, behavioral commitments, or fees during the duration of interventions would tend to make the coefficients more positive.

The findings show strong evidence that the provision of *state guarantees* is associated with enhanced systemic risk in the short run. A one-standard-deviation increase in the guarantees offered by the state normalized by total assets is linked with an increase in the systemic contribution of banks by approximately 27% of its standard deviation (as measured by *MES*). Given that the mean contribution to systemic risk is approximately 69% (i.e., the quarterly percent loss of the banks’ market capitalization during 2005–2014), the corresponding semielasticity is 17%. This finding is in line with our hypothesis that the credibility of governments’ debt guarantees was affected during the period analyzed, which could be associated with declines in bank equity values. These results can be linked with other evidence in the literature showing that guarantees from public funds can severely distort financial stability. This might happen due to the constraints on bailout budgets imposed by tight fiscal requirements or due to the lack of credibility related to their efficiency (Honohan and Klingebiel 2003; Laeven and Valencia 2012; Allen et al. 2015).

*Recapitalizations* also relate positively to banks’ systemic importance, but the effect is not significant. A possible explanation is that even though recapitalizations improve bank solvency, they also dilute the preintervention shareholders’ earnings and voting rights in most cases, which is likely to make the coefficients more positive. Additionally, the restrictions associated with recapitalizations are more severe than those involved in other bailouts (i.e., dividend bans, government interference in bank management), and the regulatory fees are higher, which can reduce the market valuation of the rescued banks.^33^

*Liquidity injections* are linked positively to systemic risk, but the effect is not notable in the short run. Usually, the liquidity lines have a window during which banks can benefit from the support; thus, they take longer to produce any effects. Additionally, they were given at lower stages of distress (i.e., a higher level of capitalization) than other types of financial aid (Berger et al. 2022) and in later rounds, as supplementary assistance after recapitalizations or state guarantees were already in place (Brei and Gadanecz 2012), which might explain why they do not carry so much weight in the short run.

The inverse Mills ratio is highly significant, which indicates that selection is present with respect to interventions. Among bank characteristics, the findings suggest that banks’ size, leverage, return on assets, and net noninterest margin are key drivers of their systemic risk contribution, in line with the findings from previous literature (Adrian and Brunnermeier 2016; Girardi and Ergün 2013; Anginer et al. 2014a). For banking market characteristics, the capital regulatory index enters the specifications with a negative sign, suggesting that tight prudential regulations regarding initial and overall capital held by banks help decrease their systemic importance.

#### Long-term effects

In Table 5 Panel C, we analyze the long-run impact of policy interventions, considering that bailouts take the same value from one quarter before the event to the quarter in which they are paid back. The repayment period varies across banks, from one year to six years. The empirical results presented in our benchmark model (Table 5 Column (7)) reflect a positive effect of *state guarantees* in the long run, but the associated coefficient is not statistically significant. This might be possible because debt guarantee support was sometimes difficult to trust. Many European governments encountered a deterioration in fiscal capacity throughout the period analyzed in Europe, which affected the credibility of their guarantees of debt issued by banks.

**Table 5:** Short-term and long-term impact of policy interventions by banks on systemic risk. This table reports the results of the *Heckman Selection Model.* Panel A depicts the first stage results from a *Probit model* used to estimate the probability of bank i to receive policy interventions in quarter t, using the following regression: *Ln(P*_*Intervened ij,t*_* /(1—P*_*Intervened ij,t*_*))* = *β*_*0*_ + *Ω* × *Identifying restrictions*_*ij,t-1*_ + *Φ* × *Bank controls*_*ij,t-1*_ + *Ψ* × *Market & Macro controls*_*j j,t-1*_ + *ʋ*_*t*_ + *ε*_*ij,t*_. The empirical specification includes several identifying restrictions (political stability, vote share of non-government parties, legislative and executive elections dummy, prevalence) and is run for the full sample of 83 banks from 22 European countries and the period accounts for 40 quarters during 2005–2014. Panels B, C, and D report the outcome equations of the following regression estimated via *OLS Fixed Effects*: *SystemicRisk*_*ij,t*_ = *β*_*0*_ + *β*_*1*_ × *Policy interventions*_*ij, event window*_ + *β*_*2*_ × *IMR*_*ij,t-1*_ + *Φ* × *Bank controls*_*ij,t-1*_ + *Ψ* × *Market & Macro controls*_*j,t-1*_ + *φ*_*i*_ + *ʋ*_*t*_ + *μ*_*jt*_ + *ε*_*ij,t*_. The sample is restricted to 30 banks that received interventions and control for the sample selection bias by including the inverse Mills ratio generated by the *Probit model* in column (1).The dependent variable is represented by MES and reflects the systemic risk of bank i from country j in quarter t. Panel B considers the short-run effects of policy interventions received by bank i (i.e., interventions take the same value from one quarter before the event to one quarter after the event), while Panel C the long-run effects (i.e., interventions take the same value from one quarter before the event to the quarter they unwind). Panel D includes both the short-run (i.e., interventions take the same value within the event window (t-1; t + 1)) and the long-run effects of policy interventions (i.e., interventions take the same value within the event window (t + 2; t + n)). Explanatory variables are one period lagged. All models include an unreported constant. Variables are winsorized at the 1^st^ and 99^th^ percentiles. Their definitions are given in Table 1. Different specifications include year fixed effects, bank fixed effects, or country × year fixed effects. Standard errors clustered at the bank level are reported in brackets

	A. Identification 1^st^ stage	B. Short run (t-1; t + 1) 2^nd^ stage			C. Long run (t-1; t + n) 2^nd^ stage			D. Short run (t-1; t + 1) and long run (t + 2; t + n) 2^nd^ stage		
	Probit	OLS	OLS	OLS	OLS	OLS	OLS	OLS	OLS	OLS
Dependent variable	Intervened (1)	MES (2)	MES (3)	MES (4)	MES (5)	MES (6)	MES (7)	MES (8)	MES (9)	MES (10)
Policy interventions										
Guarantees short run		6.546*** (1.838)	7.646*** (2.137)	7.534*** (2.211)				6.481*** (1.834)	7.289*** (2.180)	7.243*** (2.395)
Recapitalizations short run		4.864	3.140	2.825				2.439	1.634	0.491
		(3.574)	(3.323)	(3.216)				(3.213)	(2.945)	(2.920)
Liquidity injections short run		0.218	0.190	0.107				0.316	0.236	0.337
		(0.379)	(0.494)	(0.559)				(0.423)	(0.484)	(0.489)
Guarantees long run					1.148 (1.213)	1.282 (1.184)	0.567 (1.508)	0.781 (1.086)	0.849 (1.199)	0.115 (1.190)
Recapitalizations long run					−2.493 (2.003)	−3.049** (1.537)	−4.248* (2.148)	−4.710** (2.094)	−5.226** (2.586)	−7.522** (2.976)
Liquidity injections long run					0.876*** (0.278)	1.071*** (0.228)	1.084*** (0.182)	0.768** (0.320)	1.026*** (0.232)	0.979*** (0.212)
Identification										
Political stability	1.366*** (0.126)									
Vote share of non-government parties	0.070*** (0.011)									
Legislative & executive elections	0.050 (0.111)									
Prevalence	−0.517*** (0.071)									
Inverse Mills ratio		1.819 (14.408)	100.390*** (31.401)	102.529*** (36.462)	2.573 (13.015)	87.194*** (30.884)	98.141*** (34.015)	−0.759 (12.995)	87.061*** (31.803)	91.915** (35.878)
Bank characteristics										
Beta	0.328* (0.188)	−10.787 (7.041)	2.591 (7.704)	5.422 (8.848)	−10.540* (5.958)	−2.329 (7.653)	3.233 (8.029)	−10.122 (6.179)	−2.068 (7.631)	2.850 (8.387)
Size	0.503*** (0.033)	8.926 (9.533)	38.093*** (8.126)	38.157** (15.445)	7.512 (8.226)	34.366*** (8.042)	41.906*** (10.632)	9.925 (8.419)	34.014*** (8.300)	44.072*** (11.867)
Asset growth	0.000 (0.011)	0.062 (0.327)	0.239 (0.432)	0.227 (0.421)	0.076 (0.364)	0.178 (0.451)	0.214 (0.439)	0.038 (0.305)	0.076 (0.422)	0.121 (0.408)
Leverage	−0.032* (0.017)	−3.503** (1.619)	−3.169*** (1.199)	−4.095* (2.075)	−4.681** (1.703)	−3.154** (1.308)	−4.570* (2.270)	−3.395** (1.591)	−3.275** (1.386)	−3.726 (2.193)
Credit risk ratio	0.214*** (0.056)	4.879 (4.178)	11.235* (6.204)	11.376 (6.667)	4.836 (4.186)	9.987* (6.059)	10.729* (6.202)	2.973 (4.074)	8.738 (6.129)	8.837 (6.528)
Liquidity ratio	−0.005** (0.002)	−0.144 (0.123)	−0.352*** (0.126)	−0.348*** (0.119)	−0.092 (0.120)	−0.333** (0.131)	−0.321*** (0.108)	−0.068 (0.124)	−0.318** (0.126)	−0.311** (0.115)
Rollover risk ratio	−0.016*** (0.004)	−0.078 (0.231)	−0.681 (0.443)	−0.965* (0.479)	0.081 (0.208)	−0.542 (0.431)	−0.911* (0.446)	0.057 (0.208)	−0.487 (0.444)	−0.864* (0.470)
ROAA	0.115 (0.078)	0.380 (3.267)	3.095 (2.852)	3.148 (3.149)	1.134 (3.345)	3.250 (2.873)	3.495 (3.219)	−1.067 (3.002)	2.424 (2.795)	1.860 (3.344)
Gross loans share	0.022*** (0.004)	−0.624* (0.324)	0.643 (0.399)	1.050 (0.659)	−0.511 (0.331)	0.408 (0.400)	1.076 (0.686)	−0.748** (0.332)	0.404 (0.423)	0.809 (0.641)
Net non-interest margin	−0.007*** (0.003)	−0.226** (0.092)	−0.416*** (0.125)	−0.387*** (0.133)	−0.261*** (0.086)	−0.400*** (0.123)	−0.395*** (0.128)	−0.237** (0.091)	−0.385*** (0.129)	−0.365** (0.138)
Market and macro controls										
Competition	0.012*** (0.003)	−0.156 (0.279)	2.365*** (0.806)	−1.550** (0.704)	−0.186 (0.212)	2.229*** (0.767)	−0.948 (0.853)	−0.191 (0.261)	2.168*** (0.798)	−1.638** (0.772)
Capital regulatory index	0.033* (0.019)	−1.973** (0.836)	−6.230*** (2.279)	−13.742*** (2.137)	−2.359*** (0.582)	−5.736** (2.278)	−14.587*** (4.225)	−2.077*** (0.692)	−6.116*** (2.341)	−14.487*** (2.122)
Sovereign debt	0.008*** (0.002)	0.859*** (0.172)	−0.226 (0.495)	−0.532* (0.262)	0.828*** (0.159)	−0.228 (0.485)	−0.448 (0.342)	0.937*** (0.163)	−0.168 (0.499)	−0.306 (0.237)
Inflation	0.169*** (0.050)	1.198 (2.896)	3.116 (3.191)	−7.116* (3.848)	2.824 (2.736)	9.163*** (2.816)	−2.866 (4.464)	2.599 (2.569)	8.706*** (3.063)	−8.972** (4.280)
GDP growth	−0.117*** (0.026)	−0.037 (2.025)	−9.344*** (2.181)	8.532*** (2.174)	1.330 (1.898)	−6.599*** (2.220)	8.135*** (2.202)	1.401 (1.964)	−6.950*** (2.099)	8.792*** (2.055)
GFC crisis	−0.167 (0.250)	18.492*** (5.277)	13.669** (6.621)	13.025* (7.216)	20.077*** (5.471)	16.019** (6.573)	14.083* (7.364)	18.932*** (5.305)	15.325** (6.398)	13.958* (7.057)
SVG crisis	−0.864*** (0.179)	21.954** (8.761)	−34.044 (21.008)	−0.008 (5.223)	25.984*** (7.591)	−19.990 (19.918)	9.762* (5.498)	22.774** (8.694)	−15.794 (21.172)	−8.099 (7.380)
Year FE	YES	YES	NO	NO	YES	NO	NO	YES	NO	NO
Bank FE	NO	YES	NO	YES	YES	NO	YES	YES	NO	YES
Country × Year FE	NO	NO	YES	YES	NO	YES	YES	NO	YES	YES
Cluster	Banks	Banks	Banks	Banks	Banks	Banks	Banks	Banks	Banks	Banks
Observations	1,831	710	710	710	710	710	710	710	710	710
Number of banks	83	30	30	30	30	30	30	30	30	30
No of countries	22	22	22	22	22	22	22	22	22	22
Within R-squared		0.469	0.575	0.578	0.459	0.566	0.570	0.480	0.584	0.588
Pseudo R-squared	0.343									
Log-likelihood	-809.9									
Kleibergen-Paap F-test statistic	36.316									
Hansen J test statistic	1.556									
Hansen J p-value	0.6695									

^*^, ** and *** denote significance levels of 10%, 5% and 1%

In the longer-term periods, *recapitalizations* are associated with reduced systemic importance, but the effect is only marginally significant and present only in Models (6) and (7). A one-standard-deviation increase in equity injected by government normalized by total assets is associated with a decrease in banks’ contribution to systemic risk by 12% of its standard deviation. The corresponding semielasticity is approximately 8%. Considering that the long-term period in our framework also partly captures the realization of risk, this finding suggests that recapitalizations fix the problems created by risk realizations.

*Liquidity injections* provided by governments end up being positively and significantly associated with systemic risk, as suggested by the positive and significant coefficients. The economic effect is also meaningful. A one-standard-deviation increase in liquidity injections reported to total assets corresponds to an increase in banks’ marginal contribution to systemic risk by approximately 19% of its standard deviation (as measured by *MES*). Our estimates imply an associated semielasticity of 12%. This result indicates that the realization of distress continued at banks for which liquidity injections were in place in the long run. The financial health of these banks remained weak, and their stock returns underperformed.

In Panel D, we include both the short-run and the long-run effects in the same regression. For the short-run effects, we use the same definition of the event window (i.e., interventions take the same value within the window (t-1; t + 1)). However, for the long-term effects of policy interventions, we exclude the short-term period from the event window (i.e., interventions take the same value within the window (t + 2; t + n)). As shown by Columns (8)–(10), the coefficients are similar to those in the main findings.

In Table 6, we explore additional long-term strategies. We examine the effects of interventions after different time intervals, as it might be expected that the implications of bailouts vary in the long run. To account for these possible developments, we consider several event windows: one year (i.e., policy interventions are maintained at the same level from one quarter before the event to four quarters after the event) in Column (1), two years in Column (2), three years in Column (3), and four years in Column (4). The specifications are similar to the benchmark model from Table 5 Column (7), which includes bank-level, market, and macro controls, bank fixed effects and country × year fixed effects. In Column (5), we report the results of an empirical specification that includes in the same regression individual effects for each year, i.e., the 1^st^-year effects (t-1; t + 4), the 2^nd^-year effects (t + 5; t + 8), the 3^rd^-year effects (t + 9; t + 12), and the 4^th^-year effects (t + 13; t + 16). The results point to the same positive association of liquidity injections with banks’ contribution to systemic risk. State guarantees are not significantly associated with systemic importance over the period analyzed, while recapitalizations are significantly linked with reduced systemic importance only for the second year.

**Table 6:** Additional long-term effects. This table reports the estimation results of the following regression: *SystemicRisk*_*ij,t*_ = *β*_*0*_ + *β*_*1*_ × *Policy interventions*_*ij, event window*_ + *β*_*2*_ × *IMR*_*ij,t-1*_ + *Φ* × *Bank controls*_*ij,t-1*_ + *Ψ* × *Market & Macro controls*_*j,t-1*_ + *φ*_*i*_ + *μ*_*jt*_ + *ε*_*ij,t*_. We report the results for the benchmark model (i.e., Column (7) Table 5). Method used is *OLS Fixed Effects*. The sample is restricted to 30 banks that received interventions and control for the sample selection bias by including the inverse Mills ratio generated by the 1^st^ stage *Probit model*. The dependent variable is represented by MES and reflects the systemic risk of bank i from country j in quarter t. Policy interventions received by bank i from government j in quarter t take the same value from one quarter before the intervention to one-four years after the intervention in columns (1)-(4). Column (5) includes in the same regression individual effects for each year, i.e. the 1^st^-year effects (t-1; t + 4), the 2^nd^-year effects (t + 5; t + 8); the 3^rd^-year effects (t + 9; t + 12), and the 4^th^-year effects (t + 13; t + 16). The coefficients for bank characteristics (beta, size, asset growth, leverage, credit risk, liquidity, rollover risk, profitability, gross loans share, net non-interest margin), and market and macro control variables (competition, capital regulatory index, sovereign debt, inflation, GDP growth, GFC crisis, SVG crisis) are suppressed for brevity. Explanatory variables are one period lagged. All models include an unreported constant, country × year fixed effects and bank fixed effects. Variables are winsorized at the 1^st^ and 99^th^ percentiles. Their definitions are given in Table 1. Standard errors clustered at the bank level are reported in brackets

	1 year effects (t-1; t + 4)	2 years effects (t-1; t + 8)	3 years effects (t-1; t + 12)	4 years effects (t-1; t + 16)	1–4 years effects individually
	OLS 2^nd^ stage	OLS 2^nd^ stage	OLS 2^nd^ stage	OLS 2^nd^ stage	OLS 2^nd^ stage
Dependent variable	MES (1)	MES (2)	MES (3)	MES (4)	MES (5)
Policy interventions					
Guarantees long run	−0.391 (1.268)	−2.371 (1.761)	1.129 (1.527)	0.018 (1.401)	
Recapitalizations long run	0.234 (2.858)	−9.679*** (2.191)	−0.175 (3.150)	−2.525 (2.388)	
Liquidity injections long run	0.420 (0.307)	0.985*** (0.185)	0.872*** (0.233)	1.064*** (0.180)	
Guarantees long run 1^st^ year (t-1; t + 4)					−3.830 (2.383)
Recapitalizations 1^st^ year (t-1; t + 4)					−6.141 (3.929)
Liquidity injections 1^st^ year (t-1; t + 4)					0.870*** (0.250)
Guarantees long run 2^nd^ (t + 5; t + 8)					−1.290 (2.163)
Recapitalizations 2^nd^ year (t + 5; t + 8)					−15.202*** (3.693)
Liquidity injections 2^nd^ year (t + 5; t + 8)					1.543*** (0.467)
Guarantees 3^rd^ year (t + 9; t + 12)					2.032 (2.293)
Recapitalizations 3^rd^ year (t + 9; t + 12)					3.177 (3.687)
Liquidity injections 3^rd^ year (t + 9; t + 12)					1.884** (0.780)
Guarantees 4^th^ year (t + 13; t + 16)					0.407 (1.490)
Recapitalizations 4^th^ year (t + 13; t + 16)					−2.996 (2.759)
Liquidity injections 4^th^ year (t + 13; t + 16)					1.367*** (0.327)
Identification					
Inverse Mills ratio	102.356*** (35.700)	84.413** (33.930)	103.278*** (34.755)	98.882*** (34.363)	89.048** (34.613)
Bank characteristics	YES	YES	YES	YES	YES
Market and macro controls	YES	YES	YES	YES	YES
Bank FE	YES	YES	YES	YES	YES
Country × Year FE	YES	YES	YES	YES	YES
Cluster	Banks	Banks	Banks	Banks	Banks
Observations	710	710	710	710	710
Number of banks	30	30	30	30	30
No of countries	22	22	22	22	22
Within R-squared	0.562	0.579	0.566	0.568	0.601

^*^, ** and *** denote significance levels of 10%, 5% and 1%

#### Robustness

We check the robustness of our results by employing several strategies related to the sample, period, and methodology used.^34^

##### Sample

Some of the banks in our sample were severely hit by the European sovereign debt crisis, during which different rescue packages were provided. Additionally, the Eastern and Western EU member states were affected differently in terms of fiscal capacity. To assess the effects of the bailouts across different EU member states, we employ two empirical strategies. Table 7 Panel A presents the output for a restricted sample, where banks from countries severely affected by the European sovereign debt crisis (Cyprus, Ireland, Italy, Portugal, and Spain) are excluded. The findings linked to the short-run impact remain unaltered. For the long-run impact, liquidity injections maintain their positive and significant association with systemic risk. In Table 7 Panel B, we re-estimate the benchmark models for a sample restricted to banks with headquarters in Western Europe.^35^ As shown by Columns (3) and (4), the size and significance of the coefficients remain very similar to those in the main findings.

**Table 7:** Robustness assessment: sample. Panel A presents the results for a sample of banks from countries that were not affected by the European sovereign debt crisis. Panel B depicts the output for a sample restricted to Western European banks. Models (1) and (3) assess the short-term impact of policy interventions received by bank i from government j (i.e., interventions take the same value from one quarter before the event to one quarter after the event). Models (2) and (4) assess the long-term impact of policy interventions (i.e., interventions take the same value from one quarter before the event to the quarter they unwind). We report the results for the benchmark models (i.e., Column (4) Table 5 for short run effects, and, respectively, Column (7) Table 5 for long run effects. Method used is *OLS Fixed Effects.* The estimations control for the sample selection bias by including the inverse Mills ratio generated by the 1^st^ stage *Probit model*. The dependent variable is the MES. The coefficients for bank characteristics (beta, size, asset growth, leverage, credit risk, liquidity, rollover risk, profitability, gross loans share, net non-interest margin), and market and macro control variables (competition, capital regulatory index, sovereign debt, inflation, GDP growth, GFC crisis, SVG crisis) are suppressed for brevity. Explanatory variables are one period lagged. All models include an unreported constant, country × year fixed effects and bank fixed effects. Variables are winsorized at the 1^st^ and 99^th^ percentiles. Their definitions are given in Table 1. Standard errors (S.E.) clustered at bank level are reported in brackets

	A. Without banks from countries affected by the European sovereign debt crisis		B. Without banks from Eastern European countries	
	Short run (t-1; t + 1)	Long run (t-1; t + n)	Short run (t-1; t + 1)	Long run (t-1; t + n)
	OLS 2^nd^	OLS 2^nd^	OLS 2^nd^	OLS 2^nd^
Dependent variable	MES (1)	MES (2)	MES (3)	MES (4)
Policy interventions				
Guarantees short run	7.860** (3.306)		7.709*** (2.254)	
Recapitalizations short run	3.539 (5.586)		2.779 (3.029)	
Liquidity injections short run	−0.231 (0.664)		−0.387 (0.446)	
Guarantees long run		0.741 (2.502)		0.760 (1.504)
Recapitalizations long run		−5.776 (4.091)		−4.114* (2.301)
Liquidity injections long run		1.348*** (0.240)		1.302*** (0.171)
Identification				
Inverse Mills ratio	97.699** (34.760)	91.051** (31.647)	101.995** (37.697)	97.240** (35.862)
Bank characteristics	YES	YES	YES	YES
Market and macro controls	YES	YES	YES	YES
Bank FE	YES	NO	YES	NO
Country × Year FE	YES	YES	YES	YES
Cluster	Banks	Banks	Banks	Banks
Observations	540	540	648	648
Number of banks	19	19	23	23
No of countries	18	18	20	20
Within R-squared	0.579	0.575	0.588	0.580

^*^, ** and *** denote significance levels of 10%, 5% and 1%

##### Period

The main results reported in Table 5 show that systemic risk was significantly higher during the crisis period. To disentangle the effects of interventions on banks during the two crisis episodes covered by our dataset, we interact the policy interventions with dummy variables that reflect the duration of the crises.^36^ The crisis period started in 2008Q3 and ended in 2012Q4 and consists of two phases. The first phase of the crisis began after the Lehman Brothers collapse in 2008Q3 and continued through 2009Q4, corresponding with the intensification of global financial crisis effects in Europe (Brei et al. 2013). The second phase of the crisis ran from 2010Q1 to 2012Q4 and coincided with the European sovereign debt crisis of Greece, Ireland, Italy, Portugal, and Spain (De Santis 2014).

The results from Table 8 point to the same positive and significant association of state guarantees with systemic risk in the short run and of liquidity injections in the long run but no significant effect of recapitalizations. The long-run impact of state guarantees on systemic risk is negative. For the global financial crisis period, it becomes even more negative and significant (Model 2), suggesting that guaranteeing the debt issued by banks was an efficient intervention strategy during this period. In turn, for the sovereign debt crisis period, the long-run beneficial effect of state guarantees on systemic risk is diminished (Model 4). The difference between the findings for the GFC period and the European sovereign debt crisis period indicates that government credibility has an important role. It is possible that during the GFC, market participants had high expectations of the governments’ capacity to guarantee banks’ debt, which increased the valuation of banks by investors. In turn, during the European sovereign debt crisis, governments’ reliability in guaranteeing banks’ debt was affected. The fiscal capacity of some European states declined considerably during the period 2011–2012, leading to a downgrade in sovereign ratings and increasing skepticism among market participants.

**Table 8:** Robustness assessment: period. This table reports the estimation results of the following regression: *SystemicRisk*_*ij,t*_ = *β*_*0*_ + *β*_*1*_ × *Policy interventions*_*ij, event window*_ + *β*_*2*_ × *Policy interventions*_*ij, event window*_ × *Period*_*t*_ + *β*_*3*_ × *Period*_*t*_ + *β*_*4*_ × *IMR*_*ij,t-1*_ + *Φ* × *Bank controls*_*ij,t-1*_ + *Ψ* × *Market & Macro controls*_*j,t-1*_ + *φ*_*i*_ + *ʋ*_*jt*_ + *ε*_*ij,t*_. Panel A presents the results for the interaction of policy interventions with the global financial crisis period (GFC crisis), and, Panel B for the interaction of policy interventions with the sovereign debt crisis period (SVG crisis). Models (1) and (3) assess the short-term impact of policy interventions received by bank i from government j (i.e., interventions take the same value from one quarter before the event to one quarter after the event). Models (2) and (4) assess the long-term impact of policy interventions (i.e., interventions take the same value from one quarter before the event to the quarter they unwind). We report the results for the benchmark models (i.e., Column (4) Table 5 for short run effects, and, respectively, Column (7) Table 5 for long run effects. Method used is *OLS Fixed Effects.* The estimations control for the sample selection bias by including the inverse Mills ratio generated by the 1^st^ stage *Probit model*. The dependent variable is the MES. The coefficients for period (GFC crisis, SVG crisis), bank characteristics (beta, size, asset growth, leverage, credit risk, liquidity, rollover risk, profitability, gross loans share, net non-interest margin), and market and macro control variables (competition, capital regulatory index, sovereign debt, inflation, GDP growth) are suppressed for brevity. Explanatory variables are one period lagged. All models include an unreported constant, country × year effects and bank fixed effects. Variables are winsorized at the 1^st^ and 99^th^ percentiles. Their definitions are given in Table 1. Standard errors (S.E.) clustered at bank level are reported in brackets

	A. Global financial crisis		B. Sovereign debt crisis	
	Short run (t-1; t + 1)	Long run (t-1; t + n)	Short run (t-1; t + 1)	Long run (t-1; t + n)
	OLS 2^nd^	OLS 2^nd^	OLS 2^nd^	OLS 2^nd^
Dependent variable	MES (1)	MES (2)	MES (3)	MES (4)
Policy interventions				
Guarantees short run	6.496** (2.529)		9.179*** (2.853)	
Recapitalizations short run	0.327 (1.705)		4.757 (6.769)	
Liquidity injections short run	0.293 (0.425)		0.039 (0.609)	
Guarantees long run		1.389 (1.637)		−2.013 (1.788)
Recapitalizations long run		−2.921 (2.145)		−6.213*** (2.213)
Liquidity injections long run		0.927*** (0.250)		1.042*** (0.242)
Policy interventions × Period				
Guarantees × *Period*	3.898 (3.913)		−2.362 (3.694)	
Recapitalizations × *Period*	4.548 (7.485)		−4.969 (7.011)	
Liquidity injections × *Period*	−0.369 (0.778)		0.959 (0.639)	
Guarantees after event × *Period*		−9.361** (4.161)		3.998** (1.433)
Recapitalizations after event × *Period*		−4.950 (10.938)		3.334 (3.276)
Liquidity injections after event × *Period*		0.420 (0.805)		0.070 (0.403)
Identification				
Inverse Mills ratio	102.074** (36.601)	102.261*** (33.276)	102.468*** (36.384)	99.089** (35.815)
Period	YES	YES	YES	YES
Bank characteristics	YES	YES	YES	YES
Market and macro controls	YES	YES	YES	YES
Bank FE	YES	YES	YES	YES
Country × Year FE	YES	YES	YES	YES
Cluster	Banks	Banks	Banks	Banks
Observations	710	710	710	710
Number of banks	30	30	30	30
No of countries	22	22	22	22
Within R-squared	0.579	0.575	0.579	0.573

^*^, ** and *** denote significance levels of 10%, 5% and 1%

##### Methodology

We further check the robustness of our findings by including the lags of the dependent variable, replacing the sum of MES by its median value within a quarter, and using the Delta CoVaR as an alternative method for computing systemic risk. Additionally, we re-estimate the empirical specifications using several alternative strategies: propensity score matching, a placebo test, and the difference-in-differences approach. The results are presented in Table 9.

**Table 9:** Robustness assessment: methodology. This table presents robustness assessment for different methodologies. In Panel A we include the lags of the dependent variable. In Panel B we re-estimate the benchmark specifications using the median MES for the dependent variable. In Panel C we re-estimate the empirical models using Delta CoVaR as dependent variable. In Panel D we apply a *propensity score matching* analysis and use a sample obtained through nearest-neighbor strategy (N = 1). In Panel E we run a *placebo test* assuming that the rescue packages were provided to banks two years earlier. We assess their impact on systemic risk using a placebo sample from 2005 to 2007. In Panel F we run a *difference-in-differences* analysis. We report the results for the benchmark models (i.e., Column (4) Table 5 for short run effects, and, respectively, Column (7) Table 5 for long run effects). The coefficients for bank characteristics (beta, size, asset growth, leverage, credit risk, liquidity, rollover risk, profitability, gross loans share, net non-interest margin), and market and macro control variables (competition, capital regulatory index, sovereign debt, inflation, GDP growth, GFC crisis, SVG crisis) are suppressed for brevity. Explanatory variables are one period lagged. The estimations control for the sample selection bias by including the inverse Mills ratio generated by the 1^st^ stage *Probit model*. All models include an unreported constant, country × year effects and bank fixed effects. Variables are winsorized at the 1^st^ and 99^th^ percentiles. Their definitions are given in Table 1. Standard errors (S.E.) clustered at bank level are reported in brackets

	A. Including lagged MES		B. Median MES		C. Alternative dependent variable: CoVaR		D. Propensity Score Matching		E.Placebo test		F. Difference-in-Differences
	Short run (t-1; t + 1)	Long run (t-1; t + n)	Short run (t-1; t + 1)	Long run (t-1; t + n)	Short run (t-1; t + 1)	Long run (t-1; t + n)	Short run (t-1; t + 1)	Long run (t-1; t + n)	Short run (t-1; t + 1)	Long run (t-1; t + n)	After event
Method	OLS 2^nd^	OLS 2^nd^	OLS 2^nd^	OLS 2^nd^	OLS 2^nd^	OLS 2^nd^	OLS 2^nd^	OLS 2^nd^	OLS 2^nd^	OLS 2^nd^	DID 2^nd^
Dependent variable	MES (1)	MES (2)	MES (3)	MES (4)	CoVaR (5)	CoVaR (6)	MES (7)	MES (8)	MES (9)	MES (10)	MES (11)
Policy interventions											
Guarantees short run	4.946*** (1.709)		0.533*** (0.168)		3.458*** (1.029)		7.477*** (2.220)		0.572 (1.760)		
Recapitalizations short run	2.006 (2.671)		0.236 (0.241)		0.898 (1.785)		3.078 (3.354)		1.687 (3.321)		
Liquidity injections short run	0.346 (0.441)		0.014 (0.038)		−0.016 (0.234)		−0.072 (0.553)		0.537* (0.264)		
Guarantees long run		0.425 (1.043)		0.032 (0.109)		0.407 (0.724)		0.318 (1.721)		−0.927 (1.244)	
Recapitalizations long run		−1.984 (1.564)		−0.338** (0.151)		−1.646 (1.096)		−4.569** (2.169)		3.046 (4.047)	
Liquidity injections long run		0.652*** (0.162)		0.084*** (0.014)		0.463*** (0.067)		1.090*** (0.185)		3.631 (4.602)	
Guarantees × Post-intervention											−1.309 (1.875)
Recapitalizations × Post-intervention											−4.158 (2.747)
Liquidity injections × Post-intervention											0.693** (0.320)
Identification											
Inverse Mills ratio	65.401* (32.823)	62.363* (30.698)	8.392*** (2.608)	8.027*** (2.406)	44.962*** (15.731)	43.450*** (14.360)	104.785*** (36.548)	101.138*** (34.081)	−69.009 (59.827)	−48.305 (49.879)	100.009*** (35.138)
Lagged Y	YES	YES	NO	NO	NO	NO	NO	NO	NO	NO	NO
Bank characteristics	YES	YES	YES	YES	YES	YES	YES	YES	YES	YES	YES
Market and macro controls	YES	YES	YES	YES	YES	YES	YES	YES	YES	YES	YES
Bank FE	YES	YES	YES	YES	YES	YES	YES	YES	YES	YES	YES
Country × Year FE	YES	YES	YES	YES	YES	YES	YES	YES	YES	YES	YES
Cluster	Banks	Banks	Banks	Banks	Banks	Banks	Banks	Banks	Banks	Banks	Banks
Observations	710	710	710	710	710	710	692	692	124	124	710
Number of banks	30	30	30	30	30	30	30	30	30	30	30
No of countries	22	22	22	22	22	22	22	22	22	22	22
R-squared	0.654	0.649	0.589	0.583	0.591	0.581	0.581	0.573	0.404	0.399	0.564

^*^, ** and *** denote significance levels of 10%, 5% and 1%

Previous literature suggests that systemic risk measures can be persistent. For example, López-Espinosa et al. (2012) found a high level of persistence of systemic risk for a sample of large international banks. To account for this dynamic, we start by including the lag of the dependent variable among the regressors. The results from Panel A indicate that the positive impact of state guarantees on systemic importance in the short run and of liquidity injections in the long run is maintained. Unreported results confirm that the lagged MES carries a positive and significant coefficient, indicating a dynamic character of the banks’ contribution to systemic risk.

Next, we use an alternative method for computing the quarterly values of the systemic risk indicator, which are derived from weekly values. Instead of summing up the weekly MES values within a quarter, we use their median value. The results from Panel B confirm that the coefficients associated with the main variables of interest remain valid.

In Panel C, we employ the *Delta CoVaR (Conditional Value at Risk),* developed by Adrian and Brunnermeier (2016), as the dependent variable. In contrast with *MES, Delta CoVaR* assesses the contagion effects from a bank to the system. Each bank’s contribution to systemic risk is determined as the *VaR* of the system (i.e., the maximum possible loss of the system) conditioned on the event that each bank is at its own *VaR* level (i.e., the maximum possible loss of a bank)*.* Online Appendix 4.2 provides a detailed description of the estimations. We note that the positive link of systemic importance with state guarantees in the short run and with liquidity injections in the long run is maintained.

In Panel D, we provide the results of a *propensity score matching* analysis. We construct an artificial control group by matching each intervened bank with the nearest non-intervened bank from our sample with similar characteristics. First, we run a probit model that estimates the propensity scores of all banks using the bank-level characteristics employed in our main regressions (i.e., beta, size, asset growth, leverage, credit risk, liquidity, rollover risk, profitability, gross loans share, and net non-interest margin). Employing a nearest-neighbor matching strategy, we match each intervened bank with a non-intervened bank with the closest propensity score. Second, we rerun our main regressions using the matched samples. We observe that the size and significance of the findings remain unaltered.

In Panel E, we assume that the rescue packages were provided to banks two years earlier and use *a placebo sample* from 2005 to 2007. The coefficients associated with the policy interventions become insignificant, thus confirming the robustness of the impact.

Finally, we employ a *difference-in-differences* approach in Panel F to assess the difference between rescued banks before intervention versus rescued banks after intervention. In comparison with the long-term analysis where we examine the effects of interventions over several event windows (i.e., from one to four years), this model allows us to consider all quarters available after the bailouts took place within our dataset (which can imply a longer period). Policy interventions take the same value (i.e., the total volume of each type of bailout reported to banks’ total assets) from one quarter before the event to all quarters after the event available within our dataset. The results point to the same positive and significant association of liquidity injections with banks’ contribution to systemic risk in the long run and no significant impact of the other intervention mechanisms (Column (11)).

##### Other assessments

In unreported results, we conduct additional robustness exercises. First, we re-estimate the benchmark models (Columns (4) and (7) from Table 5) employing alternative variables for funding risk and profitability. We replace the liquidity ratio with the loans to deposits ratio (computed as net loans to total deposits and borrowings), the interbank liquidity ratio (interbank assets to interbank liabilities), and the return on assets ratio with the operating profit margin (operating profit to average total assets). Second, we change the level of clustering of the standard errors from the bank level to the bank and quarter level (two-way clustering). Third, instead of dividing the policy interventions received by bank i in quarter t by total assets of the bank in the same quarter, we compute their weight in total assets in the previous quarter before implementation (t-1). Fourth, we separately include the policy intervention variables in the empirical specifications. The (unreported) results show no important differences from the results of the benchmark regression specification, and the impact of the policy interventions on systemic risk in terms of sign, size and significance remains unaltered.

### Restrictions, policy interventions and systemic risk

Thus far, we have estimated the impact of policy measures on the “average” bank. This section presents the impact of restrictions imposed by regulators on the relation between emergency rescue actions and systemic risk. The model specification is introduced in subsection 3.3.2. We consider the following constraints: supervisory board intrusions, management pay limitations, and capital payout bans. Table 10 Panel A shows the empirical estimates for the short-run models, while Panel B shows the long-run specifications.

**Table 10:** Policy interventions by banks. Interactions with restrictions. This table reports the estimation results of the following regression: *SystemicRisk*_*ij,t*_ = *β*_*0*_ + *β*_*1*_ × *Policy interventions*_*ij, event window*_ + *β*_*2*_ × *Policy interventions*_*ij, event window*_ × *Restrictions*_*ij,t-1*_ + *β*_*3*_ × *Restrictions*_*ij,t-1*_ + *β*_*4*_ × *IMR*_*ij,t-1*_ + *Φ* × *Bank controls*_*ij,t-1*_ + *Ψ* × *Market & Macro controls*_*j,t-1*_ + *μ*_*jt*_ + *ε*_*ij,t*_. Method used is *OLS Fixed Effects*. The sample is restricted to 30 banks that received interventions and control for the sample selection bias by including the inverse Mills ratio generated by the 1^st^ stage *Probit model*. The dependent variable is represented by MES and reflects the systemic risk of bank i from country j in quarter t. Panel A shows the output for the short-term impact of policy interventions received by bank i from government j (i.e., interventions take the same value from one quarter before the event to one quarter after the event) interacted with restrictions imposed by government (i.e., supervisory board intrusions, management pay limitations, and capital payout bans). Panel B shows the output for the long-term impact of rescue measures interacted with bank risk profiles (i.e., interventions received by bank i from government j in quarter t take the same value from one quarter before the event to the quarter they unwind). We report the results for the models with country × year fixed effects (i.e., Column (3) Table 5 for short run effects, and, respectively, Column (6) Table 5 for long run effects). The coefficients for bank characteristics (beta, size, asset growth, leverage, credit risk, liquidity, rollover risk, profitability, gross loans share, net non-interest margin), and market and macro control variables (competition, capital regulatory index, sovereign debt, inflation, GDP growth, GFC crisis, SVG crisis) are suppressed for brevity. Explanatory variables are one period lagged. All models include an unreported constant and country × year fixed effects. Variables are winsorized at the 1^st^ and 99^th^ percentiles. Their definitions are given in Table 1. Standard errors clustered at bank level are reported in brackets

	Panel A. Short run (t-1; t + 1)				Panel B. Long run (t-1; t + n)			
	OLS 2^nd^	OLS 2^nd^	OLS 2^nd^	OLS 2^nd^	OLS 2^nd^	OLS 2^nd^	OLS 2^nd^	OLS 2^nd^
Dependent variable	MES	MES	MES	MES	MES	MES	MES	MES
*Restriction measure*	*Main* *results*	*Supervisory board* *intrusions*	*Management pay* *limitations*	*Capital payout* *bans*	*Main* *results*	*Supervisory board* *intrusions*	*Management pay* *limitations*	*Capital payout* *bans*
	(1)	(2)	(3)	(4)	(5)	(6)	(7)	(8)
Policy interventions								
Guarantees	7.534*** (2.211)	7.596*** (2.580)	5.952** (2.564)	4.896* (2.592)	0.567 (1.508)	1.987 (1.291)	0.609 (1.207)	0.718 (1.343)
Recapitalizations	2.825 (3.216)	0.062 (3.814)	−5.215 (6.801)	−8.422 (8.100)	−4.248* (2.148)	−1.423 (1.919)	3.905 (3.740)	14.930 (11.219)
Liquidity injections	0.107 (0.559)	3.836** (1.926)	3.977* (2.036)	4.014** (2.029)	1.084*** (0.182)	1.757*** (0.590)	1.521*** (0.480)	0.861 (0.824)
Restriction								
*Restriction*		−9.557* (5.654)	−14.151*** (4.491)	−14.412*** (4.399)		0.713 (4.449)	−9.303* (5.183)	−10.076* (5.504)
Interventions × Restriction								
Guarantees × *Restriction*		−0.613 (3.253)	5.171 (4.646)	5.745 (4.118)		−3.472*** (1.329)	−0.446 (1.606)	0.185 (1.713)
Recapitalizations × *Restriction*		4.848 (7.125)	8.430 (8.037)	10.125 (9.581)		−0.373 (2.544)	−7.939** (3.790)	−18.350 (11.451)
Liquidity injections × *Restriction*		−4.212** (1.927)	−4.393** (2.024)	−4.436** (2.012)		−0.775 (0.582)	−0.578 (0.487)	0.052 (0.790)
Identification								
Inverse Mills ratio	102.529*** (36.462)	98.237*** (32.710)	89.222*** (30.191)	92.610*** (30.280)	98.141*** (34.015)	89.048*** (33.316)	95.000*** (31.193)	88.285*** (31.971)
Bank characteristics	YES	YES	YES	YES	YES	YES	YES	YES
Market and macro controls	YES	YES	YES	YES	YES	YES	YES	YES
Country × Year FE	YES	YES	YES	YES	YES	YES	YES	YES
Cluster	Banks	Banks	Banks	Banks	Banks	Banks	Banks	Banks
Observations	710	710	710	710	710	710	710	710
Number of banks	30	30	30	30	30	30	30	30
No of countries	22	22	22	22	22	22	22	22
Within R-squared	0.578	0.579	0.582	0.581	0.570	0.567	0.564	0.567

^*^, ** and *** denote significance levels of 10%, 5% and 1%

The results suggest that all three types of regulatory restrictions mitigate the harmful effect of liquidity injections on systemic risk in the short run, as captured by the negative and strongly significant coefficients associated with the interaction between restrictions and liquidity injections. These findings support the hypothesis that investors believe that liquidity injections enhance value when regulators impose tighter restrictions, leading to an increase in stock prices and therefore a reduction in systemic risk. It is likely that regulatory restrictions are perceived as effective tools in reducing portfolio risk by assuring stricter monitoring of the banks injected with liquidity. For the other type of financial assistance programs, the results show no significant effect of the interaction coefficient between bailouts and restrictions. A possible explanation is that in the short run, investors give greater importance to restrictions when they are associated with rescue packages that quickly fix banks’ distress than they give to other interventions that may need a longer time horizon to produce their effect.

In the long run, we obtain a similar effect for supervisory board intrusions in the case of state guarantees and management pay limitations associated with recapitalizations. Seats on the supervisory board ensure stricter supervision of investment and lending practices after governments guarantee the debt of the financial institutions. This may counterbalance the lack of credibility regarding the governments’ ability to guarantee banks’ debt, softening the positive impact of state guarantees on systemic risk. In the case of recapitalizations, ceilings on executive salaries and bonuses may temper managers’ appetite for risky projects, leading to an increase in market valuation. Therefore, executive compensation limits could further enhance the long-run beneficial impact of recapitalizations on systemic risk. We find no significant effect for liquidity bailouts, which indicates that the realization of distress continued in the long run at banks injected with liquidity, regardless of the type of regulatory restrictions applied.

Overall, applying restrictions to rescued banks while interventions are in effect can be an efficient policy tool to reduce the positive association of interventions with systemic risk. One limitation of our framework is that the interventions that we assess are specific to the global financial crisis and the sovereign debt crisis that affected European banks. Since then, a number of regulatory and legal changes have been implemented. For example, there have been several decisions by the European Commission that imposed stricter restrictions on the rescued banks, such as dividend bans, during the duration of the bailout.^37^ Additionally, the overall legal framework changed after the adoption of the EU Bank Recovery and Resolution Directive (BRDD) in 2014.^38^ However, governments can still choose from the bailout methods that we examined to save financial institutions in distress. In future research, it will be interesting to assess the effects of policy interventions on systemic risk considering the stricter restrictions imposed on rescued banks after 2014, especially in the context of the COVID-19 pandemic.

### Risk profiles, policy interventions and systemic risk

Finally, we examine the impact of banks’ risk profiles on the relation between emergency rescue actions and systemic risk. The model specification is introduced in subsection 3.3.2. We discuss the empirical results for the following risk profile indices: size, leverage, and profitability. Table 11 Panel A shows the empirical estimates for the short-run estimates, while Panel B shows those for the long-run specifications.

**Table 11:** Policy interventions by banks. Interactions with bank risk. This table reports the estimation results of the following regression: *SystemicRisk*_*ij,t*_ = *β*_*0*_ + *β*_*1*_ × *Policy interventions*_*ij, event window*_ + *β*_*2*_ × *Policy interventions*_*ij, event window*_ × *Bank risk*_*ij,t-1*_ + *β*_*3*_ × *Bank risk*_*ij,t-1*_ + *β*_*4*_ × *IMR*_*ij,t-1*_ + *Φ* × *Bank controls*_*ij,t-1*_ + *Ψ* × *Market & Macro controls*_*j,t-1*_ + *μ*_*jt*_ + *ε*_*ij,t*_. Method used is *OLS Fixed Effects*. The sample is restricted to 30 banks that received interventions and control for the sample selection bias by including the inverse Mills ratio generated by the 1^st^ stage *Probit model*. The dependent variable is represented by MES and reflects the systemic risk of bank i from country j in quarter t. Panel A shows the output for the short-term impact of policy interventions received by bank i from government j (i.e., interventions take the same value from one quarter before the event to one quarter after the event) interacted with bank risk profiles (dummy Size, dummy Leverage, dummy Profitability). Panel B shows the output for the long-term impact of rescue measures interacted with bank risks (i.e., interventions received by bank i from government j in quarter t take the same value from one quarter before the event to the quarter they unwind). We report the results for the model with country × year fixed effects (i.e., Column (3) Table 5 for short run effects, and, respectively, Column (6) Table 5 for long run effects). The coefficients for bank characteristics (beta, size, asset growth, leverage, credit risk, liquidity, rollover risk, profitability, gross loans share, net non-interest margin), and market and macro control variables (competition, capital regulatory index, sovereign debt, inflation, GDP growth, GFC crisis, SVG crisis) are suppressed for brevity. In the model with dummy Size, we exclude the continuous Size variable. In the model with dummy Leverage, we exclude the continuous Leverage variable. In the model with dummy Profitability, we exclude the continuous ROAA variable. Explanatory variables are one period lagged. All models include an unreported constant and country × year fixed effects. Variables are winsorized at the 1^st^ and 99^th^ percentiles. Their definitions are given in Table 1. Standard errors clustered at bank level are reported in brackets

	Panel A. Short run (t-1; t + 1)				Panel B. Long run (t-1; t + n)			
	OLS 2^nd^	OLS 2^nd^	OLS 2^nd^	OLS 2^nd^	OLS 2^nd^	OLS 2^nd^	OLS 2^nd^	OLS 2^nd^
Dependent variable	MES	MES	MES	MES	MES	MES	MES	MES
*Bank risk*	*Main results*	*Size*	*Leverage*	*Profitability*	*Main results*	*Size*	*Leverage*	*Profitability*
	(1)	(2)	(3)	(4)	(5)	(6)	(7)	(8)
Policy interventions								
Guarantees	7.534*** (2.211)	6.312*** (2.339)	9.358*** (2.992)	6.980** (2.978)	0.567 (1.508)	1.517 (1.838)	1.216 (1.707)	1.194 (1.190)
Recapitalizations	2.825 (3.216)	−1.633 (3.090)	7.987* (4.227)	2.820 (3.972)	−4.248* (2.148)	−2.411 (2.343)	−3.645** (1.824)	−6.340*** (1.967)
Liquidity injections	0.107 (0.559)	1.224 (1.009)	0.058 (0.506)	0.096 (0.539)	1.084*** (0.182)	0.074 (0.552)	1.217*** (0.222)	1.056*** (0.238)
Bank risk								
*Bank risk*_*t-1*_		−1.408 (11.871)	6.899* (3.768)	−2.800 (4.405)		0.995 (16.656)	3.667 (4.635)	−8.451* (4.794)
Interventions × Bank risk								
Guarantees × *Bank risk*_*t-1*_		8.784 (6.061)	−1.696 (4.104)	1.374 (3.619)		−1.540 (2.154)	0.168 (1.879)	1.250 (0.938)
Recapitalizations × *Bank risk*_*t-1*_		16.117* (8.870)	−19.833*** (5.531)	2.438 (5.047)		−2.178 (3.656)	2.110 (2.205)	8.801*** (2.367)
Liquidity injections × *Bank risk*_*t-1*_		−1.448 (1.118)	0.635 (1.246)	0.359 (0.761)		1.225** (0.576)	−0.939* (0.486)	0.029 (0.256)
Identification								
Inverse Mills ratio	102.529*** (36.462)	98.539*** (29.884)	95.307*** (31.811)	101.051*** (31.157)	98.141*** (34.015)	90.900*** (28.997)	91.921*** (29.313)	93.212*** (28.130)
Bank characteristics	YES	YES	YES	YES	YES	YES	YES	YES
Market and macro controls	YES	YES	YES	YES	YES	YES	YES	YES
Country × Year FE	YES	YES	YES	YES	YES	YES	YES	YES
Cluster	Banks	Banks	Banks	Banks	Banks	Banks	Banks	Banks
Observations	710	710	710	710	710	710	710	710
Number of banks	30	30	30	30	30	30	30	30
No of countries	22	22	22	22	22	22	22	22
Within R-squared	0.578	0.587	0.583	0.575	0.570	0.567	0.567	0.571

^*^, ** and *** denote significance levels of 10%, 5% and 1%

Regarding size, the findings presented in Panel A Column (2) show that in the short run, the influence of recapitalizations is significantly different (although only marginally so) for larger banks in comparison to smaller banks, as suggested by the coefficient on the interaction term *Recapitalizations* × *Dummy Size* (i.e., 16.117*). For the average bank, the positive link of recapitalizations with systemic risk becomes significant, and it is increasing in size for larger banks. A possible explanation is that the market valuation of large banks injected with capital is penalized more because of the dilution of preintervention shareholders’ earnings, which translates into an increase in systemic risk. In the long run, the positive association of liquidity injections with systemic risk is even more pronounced for large banks (Panel B Column (5)). The coefficient on the interaction term *Liquidity injections* × *Dummy Size* is positive and significant (i.e., 1.225**). This result is consistent with the literature on moral hazard embedded in government support programs for TBTF banks. Large banks are usually more focused on investment activities that are riskier than traditional lending activities; thus, rescue packages may incentivize them to increase portfolio risk, which enhances their systemic importance. In terms of policy implications, these findings support the restrictions suggested by the European Commission, which requested the downsizing of several large European banks that received bailouts during the crisis.

The link between interventions and systemic risk is also related to leverage in both the short and long run. The estimates show that the immediate influence of recapitalizations is negative for better capitalized banks (Column (3)). The interaction of *Dummy leverage* with recapitalizations enters the specifications with a negative sign (i.e., -19.833***).^39^ The finding suggests that the contribution to systemic risk of banks injected with capital is lower immediately after the intervention for better capitalized banks than for less capitalized banks. The long-run results highlight that the realization of distress continues in the long run for banks injected with liquidity but is diminished when banks have a higher level of capitalization (Column (7)). These findings suggest that the provision of liquidity assistance should be oriented toward safer banks. This is in line with the actions of European governments, which provided liquidity assistance to safer financial institutions with regulatory capital above the minimum required threshold. They did not wait to intervene until bank capitalization deteriorated significantly, as it would have been costlier.

Finally, performance can be significantly associated with the relationship between recapitalizations and systemic importance in the long run, as shown by the interaction term *Recapitalizations* × *Dummy Profitability* in Column (8), which is positive and highly significant (i.e., 8.801***). The result indicates that recapitalizations fix the problems created by risk realizations, and the result is amplified for less profitable banks. In turn, for banks with higher performance, recapitalizations are linked with an increase in systemic risk. A possible explanation for this finding could be that more profitable banks are likely to borrow more and engage in risky operations, increase their portfolio risk, and therefore intensify their systemic importance.

Overall, the results suggest that the relation between policy interventions and systemic contribution varies significantly with the risk profiles of banks. Characteristics such as size, leverage, and profitability can significantly shape the relationship between bailouts and banks’ systemic importance in the long run, while the immediate link between governmental assistance programs and systemic risk is heterogeneous among banks with different sizes and levels of leverage. Most importantly, from supervisors’ perspective, the efficiency of emergency rescue measures can be mitigated or enhanced by banks’ risk strategies.

## Conclusion

In this paper, we investigate how policy interventions are associated with banks’ contribution to systemic risk in the short and long run. Using a unique bank-level dataset that consists of 83 banking institutions from 22 European countries, we estimate systemic risk based on the loss generated by the reduction in banks’ market capitalization under extreme events, employing Acharya et al.’s *MES* (2017), a measure that reflects both risk realizations and forward-looking risk. The estimations are performed for the 2005–2014 period.

Analyzing a large and original bank-level dataset on policy interventions, we then show that the bailouts are associated with different evolutions of systemic risk in the short and in the long run. We employ the Heckman selection approach and control for a variety of bank, market, and macro characteristics. In the first stage, we use a *Probit* model to estimate the probability of a bank experiencing an intervention. In the second stage, we examine the association of policy interventions with systemic risk using an *OLS fixed effects* model.

Our findings provide evidence that in the short run, banks that receive policy interventions are linked with an enhanced contribution to systemic risk, indicating a delayed effect of bailouts in fixing systemic distress. As interventions are usually applied after risk is realized, the declines in market valuation of rescued banks continue after the bailouts are implemented. The effect is strongly significant for state guarantees and might be explained by the credibility issues faced by European governments. In the long run, banks injected with liquidity remained weak, and investors penalized their stock returns, as reflected in a higher systemic contribution, while recapitalizations fixed the problem created by risk realizations.

We further provide empirical evidence that policy interventions relate differently with systemic risk across banks when regulatory restrictions are imposed and where bank risk strategies differ. In sum, the picture that arises is one in which the positive association of *guarantees* with systemic risk is weaker when the regulator appoints members to the supervisory board in the long run. The immediate influence of *recapitalizations* is negative for small and better capitalized banks, while in the long run, recapitalizations are associated with reduced systemic importance, especially for less profitable banks and in cases in which the regulator limits management pay. In the short run, injecting liquidity can reduce systemic risk when the regulator imposes restrictions such as supervisory board intrusions, management pay limitations, and capital payout bans. In the long run, the positive link between *liquidity injections* and systemic risk is mitigated for small or better capitalized banks.

Our findings suggest that banks’ regulatory restrictions and risk profiles should play a key role in the design of optimal financial assistance programs. The effectiveness of policy interventions can be significantly altered by regulatory constraints and banks’ risk strategies.

## Supplementary Information

Below is the link to the electronic supplementary material.

Supplementary file1 (DOCX 150 KB)

## Data Availability

The raw data required to reproduce the above findings are available upon request.

## Footnotes

1 We use the following terms as synonyms: policy interventions, public interventions, bailouts, financial assistance programs, and emergency rescue measures.

2 Broadly, bailouts reflect the financial support extended to a company or a country facing financial difficulties and may come in various forms. In our paper, bailouts represent the financial assistance provided by governments and central banks to financial institutions and take the form of guarantees, recapitalizations, and liquidity injections.

3 In May 2010, the European Union member states set up the European Financial Stability Facility (EFSF) that has a maximum lending capacity of 440 billion euro and the European Financial Stabilization Mechanism (EFSM) with a lending capacity of 60 billion euro. Funds are raised through borrowing from financial markets and are guaranteed by the European Commission with the European Union budget as collateral. On September 27^th^, 2012, these two funds were replaced by the European Stability Mechanism (ESM) with the aim of providing instant financial assistance to Eurozone members with a limit of 500 billion euro (ESM Annual Report 2013).

4 We use the following terms as synonyms: contribution to systemic risk, systemic contribution and (at times) systemic importance.

5 The EFSF, EFSM and ESM funds, the EU's Balance of Payments program (BoP), and bilateral loans from the International Monetary Fund (IMF), World Bank, European Investment Bank (EIB) and European Bank for Reconstruction and Development (EBRD).

6 In different exercises, we use standard errors clustered at country level, bank × year level, and bank × quarter level and two-way clustering by bank and quarter. The (unreported) results confirm that the significance of the main regressors is unaffected.

7 To alleviate the concern that countries with more connections may be countries with fewer people, Braun and Raddatz (2010) derive the probability that a former politician later sits on a bank’s board by assuming that the connections are selected randomly with replacement from a common pool. This probability gives the expected number of political connections within a country.

8 We use the term “pseudo” because we do not know what would have happened with systemic risk in the absence of the interventions.

9 We also employ the Conditional Value at Risk indicator of Adrian and Brunnermeier (2016) as a robustness check.

10 The theoretical research featuring the widely used Value at Risk indicator was initiated by Jorion (1997), Dowd (1998) and Saunders (1999). Technically, this loss “is found in the left tail” of the returns’ distribution function of the market capitalization and involves the estimation of the next loss functions: $Prob\left(R_{Market\;Equity,t}^{sys}\;\leq VaR_t^{sys}\right)=\alpha$.

11 As Fig. 2 shows, our systemic risk measure is persistent over time. A possible way to address this issue is to include the lagged dependent variable among the regressors. However, combining the lagged dependent variable with bank fixed effects in the same regression may generate biased estimated coefficients (Nickell 1981; Flannery and Hankins 2013). Therefore, we decide to follow the common practice in the literature and omit the lagged dependent variable. In a robustness check detailed in subsection 5.1.3. Robustness, we account for the persistent nature of systemic risk by including the lag of the dependent variable among the regressors. The results remain very similar to the main findings.

12 Alternatively, we use event windows of one year (i.e., policy interventions take the same value from one quarter before the event to four quarters after the event), two years, three years, four years, and all periods after the intervention takes place.

13 In the remainder of the text, the expression “systemic risk is negatively influenced” is synonymous with a decrease in systemic risk and *vice versa*.

14 For example, in 2009, the European Commission introduced a directive that required EU countries to increase their protection of retail deposits to a minimum of €50,000 and then to a minimum of €100,000 by the end of 2010.

15 In different exercises, we use standard errors clustered at the country level, bank × year level, and bank × quarter level and two-way clustering by bank and quarter. The (unreported) results confirm that the significance of the main regressors is unaffected.

16 In principle, we could separate effects of interventions from pre-intervention increases in systemic risk by examining the impact of interventions during three periods: one period before the official intervention date, one short period after the official intervention date, and one long period after the official intervention date. Unfortunately, this would not help us achieve separation because information about impending interventions almost surely reached markets before the official date, and the timing of information arrival varies across banks and interventions. We have chosen to begin periods of analysis one quarter before the official date so that all or almost all information about interventions is captured.

17 For a detailed analysis of the restrictions associated with European bailouts, see Panetta et al. (2009) and Berger et al. (2022).

18 The European governments provided bailouts to viable financial institutions. However, the likelihood of bailouts was higher with decreasing capitalization, as our empirical results show in Section 4. The motivation for this decision was to restore confidence in the financial system, stimulate the real economy, encourage interbank lending, or prevent competitive disadvantages from banks from the same country or from other countries.

19 To avoid multicollinearity issues, in the model with the dummy Size variable, we exclude the continuous Size variable. In the model with the Leverage dummy, we exclude the continuous Leverage variable. In the model with the Profitability dummy, we exclude the continuous ROAA variable.

20 For example, several banks from Greece that received government interventions are in this situation and are excluded from the final sample.

21 For the assessment of robustness, we re-estimate the empirical specifications for an extended sample of 110 banks, respecting all initial inclusion criteria while also allowing banks of smaller size with total assets between 178 million euros and 1 billion euros. The results are similar to our main findings.

22 We perform a two-sample t test with unequal variance.

23 Across our sample, some countries opted for state guarantees (i.e., Cyprus, Sweden) and others for capital injections (i.e., Belgium, France), but countries more frequently opted for both of them. Liquidity injections were more common as standalone interventions.

24 From 2013 onward, dividend bans became mandatory for European banks that receive bailouts and are subject to a restructuring plan (EC 2013).

25 In the regression analysis, several of these variables are transformed to assure stationary behavior, as indicated by panel unit root tests. Table 1 gives the transformation formulae.

26 The Boone indicator is a measure of competition in the banking market, calculated as the elasticity of profits to marginal costs. The lower the Boone indicator is, the higher the level of competition.

27 Because these data are available only for years 2007 and 2012, we maintain their level constant for the period 2008–2011 (the 2007 level), and for the period 2013–2014 (the 2012 level). The capital regulatory index takes values from 0 (relaxed regulations) to 10 (tight regulations).

28 The rule of thumb is to use the Stock and Yogo (2005) critical values. Specific to our sample, the Stock–Yogo weak identification test critical value for 5% maximal relative bias is 16.85.

29 Because our panel specifications (2^nd^ stage) include all three policy interventions together, we have to use a single estimate of the inverse Mills Ratio from the 1^st^-stage Probit equation. Therefore, we estimate the impact of the exclusion restrictions on the probability of a bank receiving a bailout, regardless of the bailout type. In robustness exercises, we rerun Eq. (1) using as a dependent variable the probability of a bank receiving each type of bailout separately. The (unreported) results show that the relation between the exclusion restrictions and each type of bailout is similar with that in the main findings in terms of sign, size, and significance.

30 We also include the policy intervention variables separately in the empirical specifications. The (unreported) results show that the sign, size, and significance of the coefficients remain unchanged. Out of 30 rescued banks in our sample, six of them received all three types of interventions, while 14 banks were subject to two types of interventions and 10 relied on a single type of bailout. Because the correlation coefficients among the three types of bailouts are low (between 0.004 and 0.022), we report the results for the specifications that consider all types of bailouts together.

31 A limitation of our analysis is that causality cannot be established with certainty due to the lack of a natural experimental setting within our framework. To address this issue, we provide alternative explanations to the maximum extent possible.

32 The dependent variable is expressed in units of percentage loss of the banks’ market capitalization within a quarter.

33 Additional results on the effects of restrictions are presented in subsection 5.2.

34 For brevity, we present the output for the short-run benchmark model (Table 5 Column (4)), and for the long-run benchmark specification (Table 5 Column (7)). Unreported results show that the findings are similar across the other empirical specifications.

35 Due to the reduced number of interventions provided to Eastern European banks, we cannot run this empirical exercise for a sample restricted to these countries, and hence, we cannot entirely exclude the possibility that the patterns that we observe can only be found in Western Europe.

36 We estimate the following regression:

5 $${SystemicRisk}_{ij,t}=\hspace{0.17em}\beta_0\hspace{0.17em}+\hspace{0.17em}\beta_1\hspace{0.17em}\times\hspace{0.17em}{Policy\;interventions}_{ij,\;event\;window}\hspace{0.17em}+\hspace{0.17em}\beta_2\times\hspace{0.17em}{Policy\;interventions}_{ij,\;event\;windcow}\hspace{0.17em}\times\hspace{0.17em}{Period}_t\hspace{0.17em}+\hspace{0.17em}\beta_3\hspace{0.17em}\times\hspace{0.17em}{Period}_t\hspace{0.17em}+\hspace{0.17em}\beta_4\hspace{0.17em}\times\hspace{0.17em}{IMR}_{ij,t-1}\hspace{0.17em}+\hspace{0.17em}\Phi\hspace{0.17em}\times\hspace{0.17em}{Bank\;controls}_{ij,t-1}\hspace{0.17em}+\hspace{0.17em}\Psi\hspace{0.17em}\times\hspace{0.17em}{Market\;\&\;Macro\;controls}_{j,t-1}\hspace{0.17em}+\hspace{0.17em}\varphi_i\hspace{0.17em}+\hspace{0.17em}\upsilon_{jt}+\hspace{0.17em}\varepsilon_{ij,t}$$

where *Period*_*t*_ is a dummy variable that takes alternatively the value one during the global financial crisis or, respectively, during the European sovereign debt crisis. The description of the other regressors is similar to that for Eq. (2).

37 The 2013 Banking Communication of the European Commission requires banks to be subject to dividend bans after receiving state aid in the form of recapitalization (European Commission 2013).

38 Since January 1^st^, 2016, under the BRDD, banks are required to convert debt-like instruments to equity for a minimum amount of 8% (i.e., implement a bail-in) before receiving a bailout (European Commission 2014). This might shape the incentives of shareholders and creditors to monitor the risk of the financial institutions (Martino 2020).

39 In unreported exercises, we interact a dummy that reflects whether a bank has Tier 1 ratio higher than the median regulatory capital ratio of the sample with bank-level interventions. The results yield similar conclusions.

## References

[CR1] Acharya VV, Yorulmazer T (2007). Too many to fail – an analysis of time inconsistency in bank closure policies. J Financ Intermed.

[CR2] Acharya V, Yorulmazer T (2008). Cash-in-the-market pricing and optimal resolution of bank failures. Rev Financ Stud.

[CR3] Acharya VV, Engle RF, Richardson M (2012). Capital Shortfall: A new approach to ranking and regulating systemic risks. Am Econ Rev.

[CR4] Acharya V, Pedersen L, Philippon T, Richardson M (2017). Measuring systemic risk. Rev Financ Stud.

[CR5] Adrian T, Brunnermeier MK (2016). CoVaR. Am Econ Rev.

[CR6] Allen F, Carletti E, Goldstein I, Leonello A (2015). Moral hazard and government guarantees in the banking industry. J Financ Regul.

[CR7] Anginer D, Demirgüç-Kunt A, Zhu M (2014). How does competition affect bank systemic risk?. J Financ Intermed.

[CR8] Anginer D, Demirgüç-Kunt A, Zhu M (2014). How does deposit insurance affect bank risk? Evidence from the recent crisis. J Bank Finance.

[CR9] Barth JR, Caprio G, Levine R (2013). Bank regulation and supervision in 180 countries from 1999 to 2011. J Financ Econ Policy.

[CR10] Bayazitova D, Shivdasani A (2012) Assessing TARP. Rev Finan Stud 25(2):377–407

[CR11] Behn B, Haselmann R, Kick T, Vig V (2017) The political economy of bank bailouts. Working paper. Available online at https://finance.unibocconi.eu/sites/default/files/files/media/attachments/paper_v0815_Bocconi20170906145836.pdf

[CR12] Berger AN, Roman RA (2015). Did TARP banks get competitive advantages?. J Financ Quant Anal.

[CR13] Berger AN, Roman RA, Sedunov J (2020). Do bank bailouts reduce or increase systemic risk? The effects of TARP on financial system stability. J Financ Intermed.

[CR14] Berger AN, Nistor S, Ongena S, Tsyplakov S (2022) Catch, restrict, and release: the real story of bank bailouts. Swiss Finance Institute Research Paper, No. 20–45, Swiss Finance Institute

[CR15] Black L, Correa R, Huang X, Zhou H (2016). The systemic risk of European banks during the financial and sovereign debt crises. J Bank Finance.

[CR16] Bostandzic D, Pelster M, Weiß GNF (2014) Systemic risk, bank capital, and deposit insurance around the world. Working Paper. Available at SSRN: https://ssrn.com/abstract=2438693

[CR17] Braun M, Raddatz C (2010). Banking on politics: When former high-ranking politicians become bank directors. World Bank Econ Rev.

[CR18] Brei M, Gambacorta L, von Peter G (2013). Rescue packages and bank lending. J Bank Finance.

[CR19] Brei M, Gadanecz B (2012) Public recapitalisations and bank risk: Evidence from loan spreads and leverage. BIS Working Papers, No. 383

[CR20] Brown CO, Dinc IS (2005). The politics of bank failures: Evidence from emerging markets. Quart J Econ.

[CR21] Brown CO, Dinç IS (2011) Too many to fail? Evidence of regulatory forbearance when the banking sector is weak. Rev Finan Stud 24(4):1378–1405

[CR22] Brownlees C, Engle R (2017). SRISK: A Conditional Capital Shortfall measure of systemic risk. Rev Financ Stud.

[CR23] Brunnermeier MK, Oehmke M (2013) Bubbles, financial crises, and systemic risk. Handb Econ Finan 2:1221–1288

[CR24] Brunnermeier MK, Dong GN, Palia D (2020) Banks’ noninterest income and systemic risk. Rev Corp Finan Stud 9(2):229–255

[CR25] Buch CM, Krause T, Tonzer L (2019). Drivers of systemic risk: Do national and European perspectives differ?. J Int Money Financ.

[CR26] Cao J, Illing G (2008) Liquidity shortages and monetary policy. CESifo Working Paper Series 2210, CESifo Group, Munich

[CR27] Choi DB (2014). Heterogeneity and stability: Bolster the strong, not the weak. Rev Financ Stud.

[CR28] Cordella T, Yeyati E (2003). Bank bailouts: Moral hazard vs. value effect. J Financ Intermed.

[CR29] Cruz C, Keefer P, Scartascini C (2016) Database of Political Institutions Codebook, 2015 Update (DPI2015). Inter-American Development Bank. Updated version of Thorsten Beck, George Clarke, Alberto Groff, Philip Keefer, and Patrick Walsh, 2001. New tools in comparative political economy: The Database of Political Institutions." 15:1, 165–176 (September), World Bank Economic Review

[CR30] Dam L, Koetter M (2012) Bank bailouts and moral hazard: evidence from Germany. Rev Finan Stud 25(8):2343–2380

[CR31] De Santis RA (2014) The euro area sovereign debt crisis: identifying flight-to-liquidity and the spillover mechanisms. J Empir Finan 26:150–170

[CR32] Demirgüç-Kunt A, Detragiache E (2002). Does deposit insurance increase banking system stability? An empirical investigation. J Monet Econ.

[CR33] Demirgüç-Kunt A, Huizinga H (2004). Market discipline and deposit insurance. J Monet Econ.

[CR34] Demirgüç-Kunt A, Huizinga H (2010). Bank activity and funding strategies: The impact on risk and returns. J Financ Econ.

[CR35] Dowd K (1998). Beyond Value-at-Risk: The new science of risk management.

[CR36] Duchin R, Sosyura D (2014). Safer ratios, riskier portfolios: Banks’ response to government aid. J Financ Econ.

[CR37] European Commission (2009) Report on competition policy. Available online at: http://ec.europa.eu/competition/publications/annual_report/2009/en.pdf. Accessed Feb 2023

[CR38] European Commission (2013) Communication from the Commission on the application, from 1 August 2013, of State aid rules to support measures in favour of banks in the context of the financial crisis (‘Banking Communication’). Official Journal of the European Union, 2013/C 216/01

[CR39] European Commission (2014) Directive 2014/59/EU of the European Parliament and of the Council of 15 May 2014 establishing a framework for the recovery and resolution of credit institutions and investment firms. Official Journal of the European Union, L 173/190

[CR40] European Stability Mechanism (2013) Annual Report. Available online at: https://www.esm.europa.eu/publications/esm-annual-report-2013. Accessed Feb 2023

[CR41] Farhi E, Tirole J (2012). Collective moral hazard, maturity mismatch and systemic bailouts. Am Econ Rev.

[CR42] Flannery MJ, Hankins KW (2013). Estimating dynamic panel models in corporate finance. J Corp Finan.

[CR43] Freixas X, Giannini C, Hoggarth G, Soussa F, Goodhart C, Illing G (1999). Lender of last resort: A review of the literature. Financial Crises, Contagion, and the Lender of Last Resort. A Reader.

[CR44] Gerhardt M, Vander Vennet R (2017). Bank bailouts in Europe and bank performance. Financ Res Lett.

[CR45] Giannetti M, Simonov A (2013). On the real effects of bank bailouts: Micro evidence from Japan. Am Econ J Macroecon.

[CR46] Girardi G, Ergün AT (2013). Systemic risk measurement: Multivariate GARCH estimation of CoVaR. J Bank Finance.

[CR47] Gropp R, Gruendl C, Guettler A (2014). The impact of public guarantees on bank risk taking: Evidence from a natural experiment. Rev Financ.

[CR48] Heckman JJ (1979) Sample selection bias as a specification error. Econ J Econ Soc 47(1):153–161

[CR49] Homar T (2016) Bank recapitalizations and lending: a little is not enough. ERSB Working Paper Series No. 16/2016

[CR50] Honohan P, Klingebiel D (2003). The Fiscal Cost Implications of an accommodating approach to banking crises. J Bank Finance.

[CR51] Huang R, Ratnovski L (2009) Why Are Canadian banks more resilient? IMF Working Paper 09/152

[CR52] Jorion P (1997). Value-at-Risk: The new benchmark for controlling market risk.

[CR53] Kane EJ (1995). Three paradigms for the role of capitalization requirements in insured financial institutions. J Bank Finance.

[CR54] Karamysheva M, Seregina E (2020) Prudential policies and systemic risk: The role of interconnections. NRU Higher School of Economics, Moscow RU

[CR55] Kick T, Koetter M, Poghosyan T (2016). Bank recapitalization, regulatory intervention, and repayment. J Money Credit Bank.

[CR56] Koetter M, Noth F (2016) Did TARP distort competition among sound unsupported banks? Econ Inq 54(2):994–1020

[CR57] Laeven L, Valencia F (2012). The use of blanket guarantees in banking crises. J Int Money Financ.

[CR58] Laeven L, Ratnovski R, Tong H (2016). Bank size and systemic risk: Some international evidence. J Bank Finance.

[CR59] Liu W-M, Ngo P (2014). Elections, political competition and bank failure. J Financ Econ.

[CR60] López-Espinosa G, Moreno A, Rubia A, Valderrama L (2012). Short-term wholesale funding and systemic risk: A global *CoVaR* approach. J Bank Finance.

[CR61] Martino E (2020). The bail-in beyond unpredictability: Creditors’ incentives and market discipline.

[CR62] Martynova N, Ratnovski L, Vlahu R (2020). Bank profitability, leverage constraints, and risk-taking. J Financ Intermed.

[CR63] Mayordomo S, Rodriguez-Moreno M, Peña JI (2014). Derivatives holdings and systemic risk in the U.S. banking sector. J Bank Finance.

[CR64] Mishkin FS (2006). How big a problem is too big to fail? A review of Gary Stern and Ron Feldman's too big to fail: The hazards of bank bailouts. J Econ Lit.

[CR65] Myerson RB (2012). A model of moral-hazard credit cycles. J Polit Econ.

[CR66] Nickell S (1981). Biases in dynamic models with fixed effects. Econometrica.

[CR67] Panetta F, Faeh T, Grande G, Ho C, King M, Levy A, Signoretti FM, Taboga M, Zaghini A (2009) An assessment of financial sector rescue programmes. Bank of Italy, Economic Research and International Relations Area, No. 47

[CR68] Perotti E, Ratnovski L, Vlahu R (2011). Capital regulation and tail risk. Int J Cent Bank.

[CR69] Petrovic A, Tutsch R (2009) National rescue measures in response to the current financial crisis. ECB Legal Working Paper Series, No 8/July 2009

[CR70] Repullo R (2005). Liquidity, risk taking, and the lender of last resort. Int J Cent Bank.

[CR71] Saunders A (1999). Financial Institutions Management: A modern perspective.

[CR72] Stock JH, Yogo M (2005) Testing for weak instruments in linear IV regression. NBER Technical Working Paper 284. http://www.nber.org/papers/T0284. Accessed Feb 2023

[CR73] Tarashev N, Borio C, Tsatsaronis K (2010) Attributing systemic risk to individual institutions. BIS Working Papers No. 308

[CR74] Weiß GNF, Bostandzic D, Neumann S (2014). What factors drive systemic risk during international financial crises?. J Bank Finance.

[CR75] Weiß GNF, Neumann S, Bostandzic D (2014). Systemic risk and bank consolidation: International evidence. J Bank Finance.

