# Supplementary material for: The Impact of Policy Interventions on Systemic Risk across Banks
Source: J Financ Serv Res. 2023 Feb 22:1–52. Online ahead of print. doi: 10.1007/s10693-023-00404-8 (PMC9944428; doi:10.1007/s10693-023-00404-8)
Supplement: Supplementary file 1 — Supplementary file1 (DOCX 150 KB) [file 10693_2023_404_MOESM1_ESM.docx]

**APPENDICES**

**Online Appendix 1.** List of banks and their systemically importance status

| **Country** | **Bank** | **FSB list (G-SIBs)** | **EBA list** | **ECB list** |  | **Country** | **Bank** | **FSB list (G-SIBs)** | **EBA list** | **ECB list** |
| --- | --- | --- | --- | --- | --- | --- | --- | --- | --- | --- |
| Austria | BKS Bank AG |  |  |  |  |  | Unione di Banche Italiane Scpa-UBI Banca |  | * | $ |
|  | Bank für Tirol und Vorarlberg AG-BTV (3 Banken Gruppe) |  |  |  |  |  | UniCredit SpA | # | * | $ |
|  | Erste Group Bank AG |  | * | $ |  | Lithuania | Siauliu Bankas |  |  |  |
|  | Oberbank AG |  |  |  |  | Malta | Bank of Valletta Plc |  | * | $ |
|  | Raiffeisen Bank International AG |  | * | $ |  |  | FIMBank Plc |  |  |  |
|  | Österreichische Volksbanken AG |  |  | $ |  |  | HSBC Bank Malta Plc |  |  | $ |
| Belgium | KBC Groep NV/ KBC Groupe SA |  | * | $ |  | Netherlands | ING Groep NV | # | * | $ |
| Bulgaria | First Investment Bank AD |  |  |  |  |  | Van Lanschot NV |  |  |  |
| Cyprus | Bank of Cyprus Public Company Limited-Bank of Cyprus Group |  | * | $ |  | Poland | Bank Handlowy w Warszawie S.A. |  | * |  |
|  | Hellenic Bank Public Company Limited |  | * | $ |  |  | Bank BGZ BNP Paribas SA |  |  |  |
| Czech Republic | Komercni Banka |  |  |  |  |  | Bank Ochrony Srodowiska SA - BOS |  | * |  |
| Denmark | Danske Bank A/S |  | * |  |  |  | Bank BPH SA |  | * |  |
|  | Jutlander Bank A/S |  |  |  |  |  | Bank Zachodni WBK SA |  |  |  |
|  | Jyske Bank A/S (Group) |  | * |  |  |  | Getin Holding SA |  |  |  |
|  | Laan & Spar Bank A/S |  |  |  |  |  | ING Bank Slaski SA - Capital Group |  |  |  |
|  | Nordjyske Bank A/S |  |  |  |  |  | mBank SA |  |  |  |
|  | Ringkjoebing Landbobank |  |  |  |  |  | Bank Millennium |  |  |  |
|  | Spar Nord Bank |  |  |  |  |  | Bank Polska Kasa Opieki SA-Bank Pekao SA |  |  |  |
|  | Sydbank A/S |  | * |  |  |  | Powszechna Kasa Oszczednosci Bank Polski SA - PKO BP SA |  | * |  |
| Finland | Alandsbanken Abp-Bank of Aland Plc |  |  |  |  | Portugal | Banco Comercial Português SA-Millennium BCP |  | * | $ |
| France | Crédit Agricole S.A. | # | * | $ |  |  | Banco Espirito Santo SA |  | * |  |
|  | BNP Paribas | # | * | $ |  |  | Banco BPI SA |  | * | $ |
|  | Crédit Industriel et Commercial SA - CIC |  |  |  |  | Romania | BRD-Groupe Societe Generale SA |  |  |  |
|  | Société Générale SA | # | * | $ |  |  | Transilvania Bank-Banca Transilvania SA |  |  |  |
|  | Natixis SA |  |  |  |  | Slovakia | OTP Banka Slovensko a.s. |  |  |  |
| Germany | Commerzbank AG |  | * | $ |  |  | Prima Banka Slovensko a.s. |  |  |  |
|  | Deutsche Bank AG | # | * | $ |  |  | Tatra Banka a.s. |  |  | $ |
|  | Oldenburgische Landesbank - OLB |  |  |  |  |  | Vseobecna Uverova Banka a.s. |  |  | $ |
|  | UmweltBank AG |  |  |  |  | Spain | Banco Bilbao Vizcaya Argentaria SA |  | * | $ |
| Hungary | OTP Bank Plc |  | * |  |  |  | Bankinter SA |  | * | $ |
| Ireland | Allied Irish Banks plc |  | * | $ |  |  | Caixabank SA |  |  |  |
|  | Bank of Ireland-Governor and Company of the Bank of Ireland |  | * | $ |  |  | Banco Popular Espanol SA |  | * | $ |
| Italy | Banca Popolare dell'Emilia Romagna |  | * | $ |  |  | Banco de Sabadell SA |  | * | $ |
|  | Banco di Sardegna SpA |  |  |  |  |  | Banco Santander SA | # | * | $ |
|  | Banca Carige SpA |  | * | $ |  | Sweden | Nordea Bank AB | # | * |  |
|  | Banca Piccolo Credito Valtellinese-Credito Valtellinese Soc Coop |  | * |  |  |  | Skandinaviska Enskilda Banken AB |  | * |  |
|  | Intesa Sanpaolo |  | * | $ |  |  | Svenska Handelsbanken |  | * |  |
|  | Mediobanca SpA |  | * | $ |  |  | Swedbank AB |  | * |  |
|  | Banca Popolare dell'Etruria e del Lazio Soc. coop. |  |  |  |  | United Kingdom | Lloyds Banking Group Plc |  | * |  |
|  | Banca Popolare di Milano SCaRL |  | * | $ |  |  | Royal Bank of Scotland Group Plc | # | * |  |
|  | Banca Profilo SpA |  |  |  |  |  | Standard Chartered Plc | # |  |  |
|  | Banca Popolare di Spoleto SpA |  |  |  |  | Total | 83 | 10 | 44 | 32 |

# denotes that the bank is included in the Financial Stability Board (FSB) list of G-SIBs (Global Systemically Important Banks). * denotes that the bank is included in the European Banking Association (EBA) stress testing exercise. $ denotes that the bank is included in the European Central Bank (ECB) Single Supervisory Mechanism (SSM).

**Online Appendix 2.** Policy interventions implemented at bank level (events). This appendix presents bank level policy interventions data extracted from banks’ annual reports, financial statements, websites, and the State Aid Register of European Commission.

|  |  | **State guarantees** | | | | **Recapitalizations** | | | | **Liquidity injections** | | | |
| --- | --- | --- | --- | --- | --- | --- | --- | --- | --- | --- | --- | --- | --- |
| **Country** | **Bank** | **Size** (bil eur) | **Date** | **Type** | **Source** | **Size** (bil eur) | **Date** | **Type** | **Source** | **Size** (bil eur) | **Date** | **Type** | **Source** |
| Austria | Erste Group Bank AG | 4.05 | Jun//2009 | Debt instruments | Erste Bank Group Annual Report 2009 | 1.00 | Mar//2009 | Core Tier 1 participation capital | Erste Bank Group Annual Reports 2009, 2010 |  |  |  |  |
|  |  |  |  |  |  | 0.22 | May//2009 | Core Tier 1 participation capital | Erste Bank Group Annual Reports 2009, 2010 |  |  |  |  |
| Austria | Raiffeisen Bank International AG | 2.75 | Jan-Mar//09 | Debt instruments (EUR 1.5 bn in 28 Jan. 2009 and EUR 1.75 bn in 4 Mar. 2009) | Raiffeisen Annual report 2009 | 1.75 | 6-Apr-09 | Participation capital | Raiffeisen Annual report 2009 |  |  |  |  |
|  |  | 1.50 | 23-Apr-09 | Debt instruments | Raiffeisen Annual report 2009 |  |  |  |  |  |  |  |  |
| Austria | Österreichische Volksbanken AG | 2.00 | Feb-Mar//2009 | Debt instruments | EC Decision C(2015) 4635 on SA.31883 - 2015/N, 2011/C | 1.00 | Apr//2009 | Participation certificates | EC Decision C(2015) 4635 on SA.31883 - 2015/N, 2011/C |  |  |  |  |
|  |  | 1.00 | 14-Sep-09 | Debt instruments | EC Decision C(2015) 4635 on SA.31883 - 2015/N, 2011/C | 0.25 | 19-Sep-12 | Ordinary shares | EC Decision C(2015) 4635 on SA.31883 - 2015/N, 2011/C |  |  |  |  |
|  |  | 0.10 | 15-Mar-13 | Asset guarantee | EC Decision C(2015) 4635 on SA.31883 - 2015/N, 2011/C |  |  |  |  |  |  |  |  |
| Belgium | KBC Groupe SA |  |  |  |  | 3.50 | 18-Dec-08 | Core Tier-1 securities (Belgian State) | EC Decision C(2009) 5268 on C 18/2009 (ex N 360/2009) | 20.00 | 18-Jun-09 | Protection on CDO portfolio | EC Decision C(2009) 5268 on C 18/2009 (ex N 360/2009) |
|  |  |  |  |  |  | 3.50 | 18-Jun-09 | Core Tier-1 securities (Flemish Region) | EC Decision C(2009) 5268 on C 18/2009 (ex N 360/2009) |  |  |  |  |
| Bulgaria | First Investment Bank AD |  |  |  |  |  |  |  |  | 0.60 | 29-Jun-14 | State deposit received under the Liquidity Support Scheme (LSS) | EC Decision C(2014) 8959 on SA.39854 (2014/N) |
| Cyprus | Bank of Cyprus Public Company Limited | 1.00 | July//2013 | Debt instruments | Bank of Cyprus Group Annual Report 2013, 2014 |  |  |  |  | 11.40 | Apr//2013 | Emergency Liquidity Assistance (following the absorption of Laiki Bank) | European Parliament. Briefing - Cyprus' financial assistance programme (March 2016), BoC Annual Report 2013, BoC Press Release 17 August 2016 |
| Denmark | Danske Bank A/S | 4.70 | June & July 2009 | Bonds DKK 35 bil | Danske Bank Annual Report 2009 & 2011 & EC Decision C(2009) 6441 on S.A. N415/2009 and NN 46/2009 | 3.49 | May//2009 | Subordinated loan capital in the form of hybrid capital, in the amount of DKK 24 billion | Danske Bank Annual Report 2009 |  |  |  |  |
| Denmark | Spar Nord Bank |  |  |  |  | 0.17 | June//2009 | Hybrid core capital in the amount of DKK 1,265 million | Spar Nord Bank Annual Report 2009 & Risk Report 2009 |  |  |  |  |
| France | BNP Paribas |  |  |  |  | 5.10 | 27-Mar-09 | Preffered shares | EC Decision C(2008) 8278 on SA.613/2008, Les concours publics aux établissements de crédits : Bilan et enseignements à tirer, Rapport public thématiqu, Cour des comptes, May 2010 & BNP Paribas Financial Statements 2009 q2 | 1.80 | 2008q4 | Interbank loans guaranteed by Société de Financement de l’Économie Française (SFEF) of EUR 1.8 bil. | BNP Paribas Annual Report 2008 |
|  |  |  |  |  |  |  |  |  |  | 11.00 | 2009 | Interbank loans guaranteed by Société de Financement de l’Économie Française (SFEF) of EUR 11 bil. during 2009 | BNP Paribas Annual Report 2009 |
| France | Crédit Agricole S.A. |  |  |  |  | 3.00 | 8-Dec-08 | Super subordinated notes | EC Decision C(2008) 8278 on SA.613/2008 & Annual Report 2008 | 3.50 | Nov-Dec//2008 | Interbank loans guaranteed by Société de Financement de l’Économie Française (SFEF) | Crédit Agricole Annual Report 2008 |
|  |  |  |  |  |  |  |  |  |  | 21.60 | 2009 | Interbank loans guaranteed by Société de Financement de l’Économie Française (SFEF) of EUR 21.6 bil. during 2009, out of which EUR 15.1 billion were granted in the first half of 2009 | Crédit Agricole Annual Report 2009 & Interim Report 2009q2 |
|  |  |  |  |  |  |  |  |  |  | 20.00 | Jan-June//2010 | Interbank loans guaranteed by Société de Financement de l’Économie Française (SFEF) of EUR 20 bil. during first half of 2010 | Crédit Agricole Annual Report 2010 & Interim Report 2010q2 |
|  |  |  |  |  |  |  |  |  |  | 15.90 | Jan-June//2011 | Interbank loans guaranteed by Société de Financement de l’Économie Française (SFEF) of EUR 15.9 bil. during first half of 2011 | Crédit Agricole Annual Report 2011 & Interim Report 2011q2 |
| France | Natixis SA | 0.84 | Dec//2008 | Debt instruments | Natixis Annual Report 2009 | 2.00 | 26-Jun-09 | Deeply subordinated perpetual notes (Tier 1 capital) | Natixis Annual Report 2009 | 0.84 | Dec//2008 | Interbank loans guaranteed by Société de Financement de l’Économie Française (SFEF) | Natixis Annual Report 2008 |
|  |  |  |  |  |  | 3.15 | 1-Jan-10 | Deeply subordinated perpetual notes (Tier 1 capital) | Natixis Annual Report 2010 | 3.84 | Jan-June//2009 | Interbank loans guaranteed by Société de Financement de l’Économie Française (SFEF) | Natixis Interim Report 2009q2 |
|  |  |  |  |  |  | 0.80 | 1-Jan-11 | Deeply subordinated perpetual notes (Tier 1 capital) | Natixis Annual Report 2011 |  |  |  |  |
| France | Société Générale SA |  |  |  |  | 1.66 | 19-May-09 | Preferred shares | EC Decision C(2008) 8278 on SA.613/2008 & Société Générale Querterly Reports 2009 q1 & q2 | 1.80 | 2008q4 | Interbank loans guaranteed by Société de Financement de l’Économie Française (SFEF) | Société Générale Annual Report 2008 |
|  |  |  |  |  |  |  |  |  |  | 11.80 | 2009 | Interbank loans guaranteed by Société de Financement de l’Économie Française (SFEF) of EUR11.8 bil. during 2009, out of which EUR 9.2 billion were granted in the first half of 2009 ( | Société Générale Annual Report 2009 & Interim Report 2009q2 |
| Germany | Commerzbank AG | 15.00 | 7-May-09 | Guarantee for debt securities | EC Decision C(2012) 2227 on SA.34539 (2012N) | 8.20 | 31-Dec-08 | EUR 8.2bn in Silent participation (1^st^) perpetual hybrid Tier 1 capital from the Special Fund for Financial Market Stabilization (SoFFin) | EC Decision C(2012) 2227 on SA.34539 (2012N), Commerzbank Annual Reports 2008 and 2009, and Commerzbank Interim Reports 2009q1, 2009q2, 2009q3 |  |  |  |  |
|  |  |  |  |  |  | 10.00 | 4-Jun-09 | EUR 8.2bn in silent participation (2nd) - perpetual hybrid Tier 1 capital, and EUR 1.8bn in ordinary shares from the Special Fund for Financial Market Stabilization (SoFFin) | EC Decision C(2012) 2227 on SA.34539 (2012N), Commerzbank Annual Reports 2008 and 2009, and Commerzbank Interim Reports 2009q1, 2009q2, 2009q3 |  |  |  |  |
| Hungary | OTP Bank Plc |  |  |  |  |  |  |  |  | 1.40 | 26-Mar-09 | Loan facility: the bank was granted a total of EUR 500.8 million, GBP 135.9 million, JPY 20.1 billion and USD 818 million in two tranches | OTP Annual Report 2009 |
|  |  |  |  |  |  |  |  |  |  | 0.34 | Q3//2009 | EBRD provided a CHF 0.5 bil CHF/HUF swap facility | OTP Annual Report 2009 |
| Ireland | Allied Irish Banks plc | 6.00 | Dec//2009 | Bonds issue | AIB Annual Report 2009 | 3.50 | 13-May-09 | Core Tier 1 new preference shares | EC Decision C(2011) 5177 on SA.33296 (2011/N) | 8.50 | Dec//2010 | NAMA had acquired €21.3 billion of gross loans for consideration of €9.4 billion (ECB eligible, Government guaranteed senior notes) | National Asset Management Agency – Annual Report 2010 |
|  |  | 6.00 | Dec//2010 | Bonds issue | AIB Annual Report 2010 | 3.70 | 23-Dec-10 | Core Tier 1 ordinary shares | EC Decision C(2011) 5177 on SA.33296 (2011/N) | 9.40 | Dec//2011 | NAMA had acquired €18.5 billion of gross loans for consideration of €8.5 billion (ECB eligible, Government guaranteed senior notes) | National Asset Management Agency – Section 227 Review (2011) |
|  |  | 3.70 | Jan//2011 | Bonds issue | AIB Annual Report 2011 | 14.80 | 5-Jul-11 | Recapitalization provided to facilitate the merger of Allied Irish Bank and EBS, EUR 13.3 bn. were identified for AIB (EUR 11.9 bn.in common equity and EUR 1.4 bn. in contingent capital) and EUR 1.5 billion for EBS ( EUR 1.3 billion in common equity and EUR 0.2 bn. in contingent capital). | EC Decision C(2011) 5177 on SA.33296 (2011/N) |  |  |  |  |
| Ireland | Bank of Ireland-Governor and Company of the Bank of Ireland | 1.25 | Jun//2008 | Debt instruments | Bank of Ireland Annual Report 2009 | 3.50 | 26-Mar-09 | Core Tier 1 (preference stock) | State aid SA.33216 (2011/N); State aid SA.33443 (2011/N) | 5.20 | Dec//2010 | NAMA had acquired €9.4 billion of gross loans for consideration of €5.2 billion (ECB eligible, Government guaranteed senior notes) | National Asset Management Agency – Section 227 Review (2010) |
|  |  | 2.00 | Nov//2008 | Debt instruments | Bank of Ireland Annual Report 2009 | 5.20 | 31-Jul-11 | EUR 4.2 bil Core Tier 1 capital and EUR 1 bil contingent capital | State aid SA.33216 (2011/N); State aid SA.33443 (2011/N) | 5.60 | Dec//2011 | NAMA had acquired €9.9 billion of gross loans for consideration of €5.6 billion (ECB eligible, Government guaranteed senior notes) | National Asset Management Agency – Section 227 Review (2011) |
| Italy | Banca Piccolo Credito Valtellinese-Credito Valtellinese Soc Coop |  |  |  |  | 0.20 | 30-Dec-09 | Tier 1 (Tremonti bonds) | Banca Piccolo Credito Valtellinese Annual Report 2009 |  |  |  |  |
| Italy | Banca Popolare di Milano SCaRL | 1.50 | 23-Dec-11 | Bonds | BPM Annual Report 2009 | 0.50 | 4-Dec-09 | Tier 1 qualifying hybrid instruments | EC Decision C(2010) 7293 on SA.N 425/2010 |  |  |  |  |
| Italy | Intesa Sanpaolo | 12.00 | 6-Dec-11 | Bonds | Intesa Sanpaolo Annual Report 2011 |  |  |  |  |  |  |  |  |
| Italy | Mediobanca SpA | 3.50 | 31-Dec-11 | Bonds | Mediobanca Annual Report 2012 |  |  |  |  |  |  |  |  |
| Italy | Unione di Banche Italiane Scpa-UBI Banca | 6.00 | 2-Jan-12 & 27-Feb-12 | Bonds | UBI Annual Reports 2012, 2013 |  |  |  |  |  |  |  |  |
| Netherlands | ING Groep NV | 12 | Q1//2009 | Bonds | ING Bank Annual Report 2009 | 10.00 | 12-Nov-08 | Core Tier 1 securities | EC Decision C(2012) 8238 on SA.33305 (2012C) and SA.29832 (2012C) | 5.00 | 31-Mar-09 | ‘Illiquid Assets Back-up Facility (IABF) - cash flow swap. ING transferred 80% of the economic ownership of its Alt-A portfolio to the Dutch State. The transaction price was 90% of the par value with respect to the 80% proportion of the portfolio of which the Dutch State has become the economic owner. European Commission calculated the amount of state aid as the difference between the transfer price (the amount of the liability contracted by the state, EUR 21.6 billion) and the market value (EUR 16.6 billion), concluding that the amount of state aid associated with the IABF amounted to EUR 5 billion. | EC State aid C 10/2009 (ex N 138/2009), EC/09/1729, EC IP/09/514, ING Bank Annual Report 2009, ING Press Release 26 January 2009 |
| Portugal | Banco BPI SA |  |  |  |  | 1.50 | 29-Jun-12 | Contingent convertible subordinated bonds ("CoCos") | EC Discusion C(2013) 4802 on SA.35238 (2013N) |  |  |  |  |
| Portugal | Banco Comercial Português, SA-Millennium bcp | 1.50 | 9-Jan-09 | Bond issue guarantee | Millenium BCP Annual Report 2009 | 3.00 | 29-Jun-12 | Contingent convertible subordinated bonds ("CoCos") | EC Discusion C(2013) 5669 on SA.34724 (2013N) |  |  |  |  |
|  |  | 1.75 | Q3//2011 | Bond issue guarantee | Millenium BCP Annual Report 2011 |  |  |  |  |  |  |  |  |
|  |  | 2.90 | 20-Feb-12 | Bond issue guarantee | Millenium BCP Annual Report 2012 |  |  |  |  |  |  |  |  |
|  |  | 0.25 | 1-Jan-14 | Bond issue guarantee | EC Discusion C(2013) 5669 on SA.34724 (2013N) |  |  |  |  |  |  |  |  |
| Portugal | Banco Espirito Santo SA | 1.50 | 9-Jan-09 | Bond issue guarantee | BES Annual Report 2009 |  |  |  |  |  |  |  |  |
|  |  | 1.25 | 19-Jul-11 | Senior notes guarantee | BES Annual Report 2011 |  |  |  |  |  |  |  |  |
|  |  | 1.00 | 23-Dec-11 | Bond issue guarantee | BES Annual Report 2011 |  |  |  |  |  |  |  |  |
|  |  | 2.50 | 6-Jan-12 | Debt guarantee | BES Annual Report 2012 |  |  |  |  |  |  |  |  |
| Spain | Banco de Sabadell SA | 3.26 | 30-Dec-08 | Bond issue guarantee (including those of Banco Guipuzcoano) | Banco de Sabadell Annual Report 2008 | 2.45 | June//2012 | Capital injection by CIDGF as part of the process of acquiring Caja de Ahorros del Mediterráneo (CAM). On 1 June 2012 Banco Sabadell completed the acquisition of 100% of the shares of Banco CAM. | Banco de Sabadell Annual Report 2011 & Banco de Espana reports | 24.66 | Jun//2012 | Asset protection scheme (APS) under which the Deposit Guarantee Fund (DGF) as part of the process of acquiring Caja de Ahorros del Mediterráneo (CAM). On 1 June 2012 Banco Sabadell completed the acquisition of 100% of the shares of Banco CAM. | Banco de Sabadell Annual Report 2011 |
|  |  | 2.05 | 30-Sep-09 | Bond issue guarantee (including those of Banco Guipuzcoano) | Banco de Sabadell Annual Report 2009 | 0.25 | Oct//2013 | Subscription of capital by FROB (Fondo de Reestructuracio Ordenada Bancaria) to Banco Gallego as part of the process of being acquired by Banco de Sabadell. On October 28, 2013 the Bank took control of the business. |  |  |  |  |  |
| Spain | Caixabank, S.A. |  |  |  |  | 0.98 | 1-Jul-12 | Subscription of preference shares by FROB (Fondo de Reestructuracio Ordenada Bancaria) in Banca Cívica group prior to integration in Caixagroup (July 1, 2012). | Banco de Espana. *Background note on the public financial assistance in the recapitalisation of the Spanish banking system (2009-2013)*, 02.09.2013 | 6.42 | 27-Nov-12 | FROB Annual Report 2016**:** On 27 November 2012, the FROB arranged an APS for the buyer of Banco Valencia (CaixaBank), maturing on 30 September 2022, covering 72.5% of any losses on a closed loan book, which initially totalled EUR 6.424 billion, with a first-loss threshold of EUR 402 million. | Caixabank Annual report 2012 & FROB Annual Report 2016 |
|  |  |  |  |  |  | 4.50 | Dec//2012 | Subscription of capital by FROB (Fondo de Reestructuracio Ordenada Bancaria) in Banco de Valencia prior to integration in Caixagroup . On February 28, 2013, CaixaBank finalized the acquisition of the FROB’s 98.9% stake in Banco de Valencia, having obtained the necessary authorizations and after payment by the FROB, in December, of €4,500 million for a capital increase. |  |  |  |  |  |
| Sweden | Swedbank AB | 12.24 | 29-Nov-09 | Debt issue SEK 126 bn (about 12.24 bil eur) | Swedbank Annual Report 2008 |  |  |  |  |  |  |  |  |
|  |  | 11.45 | 2009q1 | Debt issue SEK 119 bn (about 11.45 bil eur) | Swedbank Interim Report 2009q1 |  |  |  |  |  |  |  |  |
|  |  | 7.60 | 2009q2 | Debt issue SEK 79 bn (about 7.60 bil eur) | Swedbank Interim Report 2009q2 |  |  |  |  |  |  |  |  |
|  |  | 0.09 | 2009q3 | Debt issue SEK 1 bn (about 0.10 bil Eur) | Swedbank Interim Report 2009q3 |  |  |  |  |  |  |  |  |
| United Kingdom | Lloyds Banking Group Plc | 60.00 | 31-Dec-09 | Debt issue £ 49 bn | Lloyds Annual Report 2009 | 19.00 | 19-Jan-09 | Capital injection of GBP 13bn in ordinary shares and GBP 4bn in preference shares | EC Decision C(2009) 9087 on SA.N 428/2009 | 188.00 | 21-Apr-08 | £157 billion from BoE swap temporarily illiquid assets for treasury bills through Special Liquidity Scheme (SLS) | Lloyd's Annual Report 2009 |
|  |  |  |  |  |  | 6.25 | 3-Nov-09 | Capital injection of GBP 5.9bn (rights issue) | EC Decision C(2009) 9087 on SA.N 428/2009 | 312.00 | 7-Mar-09 | Asset Protection Scheme (APS), asset covered £ 260 bn | EC Decision C(2009) 9087 on SA.N 428/2009 |
| United Kingdom | Royal Bank of Scotland Group Plc | 40.00 | 31-Dec-08 | Debt issue £ 32.2 bn | RBS Annual Report 2008 | 22.90 | 1-Dec-08 | Capital injection of GBP 15bn in ordinary shares and GBP 5bn in preference shares | EC Decision C(2009) 10112 on SA.N 422/2009 and SA.N 621/2009 | 45.00 | 17-Oct-08 | £ 36.6 bn from BoE to swap temporarily illiquid assets for treasury bills through Special Liquidity Scheme (SLS) | EC Decision C(2009) 10112 on SA.N 422/2009 and SA.N 621/2009 |
|  |  | 21.70 | 31-Dec-09 | Debt issue £ 19.3 bn | RBS Annual Report 2009 | 28.00 | 3-Nov-09 | Capital injection of GBP 25.5bn in non-voting B shares | EC Decision C(2009) 10112 on SA.N 422/2009 and SA.N 621/2009 | 215.00 | 22-Dec-09 | Asset Protection Scheme (APS), asset covered £ 282 bn | RBS Annual Report 2009 |

**Online Appendix 3.** Summary of policy interventions implemented at bank level during 2008-2014. This appendix presents the summary of bank level policy interventions data extracted from banks’ annual reports, financial statements, websites, and the State Aid Register of European Commission.

| **Country** | **Bank** | **State guarantees** | | | **Recapitalizations** | | | **Liquidity injections** | | |
| --- | --- | --- | --- | --- | --- | --- | --- | --- | --- | --- |
|  |  | No. events | Total injection size  (bil. Eur) | Average injection size (%Total Assets) | No. events | Total injection size  (bil. Eur) | Average injection size (%Total Assets) | No. events | Total injection size  (bil. Eur) | Average injection size (%Total Assets) |
| Austria | Erste Group Bank AG | 1 | 4.05 | 1.98% | 2 | 1.22 | 0.31% |  |  |  |
|  | Raiffeisen Bank International AG | 2 | 4.25 | 2.68% | 1 | 1.75 | 2.25% |  |  |  |
|  | Österreichische Volksbanken AG | 3 | 3.10 | 6.44% | 2 | 1.25 | 2.61% |  |  |  |
| Belgium | KBC Groep NV |  |  |  | 2 | 7.00 | 1.00% | 1 | 20.00 | 5.81% |
| Bulgaria | First Investment Bank AD |  |  |  |  |  |  | 1 | 0.60 | 13.02% |
| Cyprus | Bank of Cyprus Public Company Li | 1 | 1.00 | 3.19% |  |  |  | 1 | 11.40 | 34.59% |
| Denmark | Danske Bank A/S | 1 | 4.70 | 1.06% | 1 | 3.49 | 0.80% |  |  |  |
|  | Spar Nord Bank |  |  |  | 1 | 0.17 | 1.98% |  |  |  |
| France | BNP Paribas |  |  |  | 1 | 5.10 | 0.25% | 5 | 12.80 | 0.12% |
|  | Crédit Agricole S.A. |  |  |  | 1 | 3.00 | 0.18% | 9 | 61.00 | 0.41% |
|  | Natixis SA |  |  |  | 3 | 5.95 | 0.43% | 2 | 4.68 | 0.46% |
|  | Société Générale SA |  |  |  | 1 | 1.66 | 0.16% | 5 | 13.60 | 0.25% |
| Germany | Commerzbank AG | 1 | 15.00 | 1.65% | 2 | 18.20 | 1.20% |  |  |  |
| Hungary | OTP Bank Plc |  |  |  |  |  |  | 1 | 1.40 | 4.30% |
| Ireland | Allied Irish Banks plc | 3 | 15.70 | 3.37% | 3 | 22.00 | 5.39% | 2 | 17.90 | 6.37% |
|  | Bank of Ireland | 2 | 3.25 | 0.82% | 2 | 8.70 | 2.57% | 2 | 10.80 | 3.36% |
| Italy | Banca Piccolo Credito Valtelline |  |  |  | 1 | 0.20 | 0.80% |  |  |  |
|  | Banca Popolare di Milano SCaRL | 1 | 1.50 | 2.89% | 1 | 0.50 | 1.13% |  |  |  |
|  | Intesa Sanpaolo | 1 | 12.00 | 1.88% |  |  |  |  |  |  |
|  | Mediobanca SpA | 1 | 3.50 | 4.80% |  |  |  |  |  |  |
|  | Unione di Banche Italiane Scpa-U | 1 | 6.00 | 4.56% |  |  |  |  |  |  |
| Netherlands | ING Groep NV | 1 | 12.00 | 0.94% | 1 | 10.00 | 0.75% | 1 | 5.00 | 0.39% |
| Portugal | Banco BPI SA |  |  |  | 1 | 1.50 | 3.36% |  |  |  |
|  | Banco Comercial Português, SA-Mi | 4 | 6.40 | 1.70% | 1 | 3.00 | 3.23% |  |  |  |
|  | Banco Espirito Santo SA | 4 | 6.25 | 1.95% |  |  |  |  |  |  |
| Spain | Banco de Sabadell SA | 2 | 5.31 | 3.28% | 2 | 2.69 | 0.81% | 1 | 24.66 | 14.81% |
|  | Caixabank, S.A. |  |  |  | 2 | 5.48 | 0.79% | 1 | 6.42 | 1.85% |
| Sweden | Swedbank AB | 4 | 31.38 | 4.70% |  |  |  |  |  |  |
| United Kingdom | Lloyds Banking Group Plc | 1 | 60.00 | 5.20% | 2 | 25.25 | 2.35% | 1 | 188.00 | 40.46% |
|  | Royal Bank of Scotland Group Plc | 2 | 61.70 | 1.36% | 2 | 50.90 | 1.19% | 2 | 260.00 | 6.53% |
|  | **Total** | **36** | **257.09** | **2.87%** | **35** | **179.02** | **1.52%** | **35** | **638.26** | **8.85%** |

**Online Appendix 4. Systemic risk measures**

This appendix provides a detailed description of the methodologies that we use to estimate the *Marginal Expected Shortfall (MES)* and the *Conditional Value at Risk (CoVaR)*. The measures are estimated on a weekly basis and then summed up within a quarter. In our framework, we assume that the reduction in a bank’s market capitalization below a target level imposes external costs on the system during distress and generates an increased contribution of the bank to systemic risk. This hypothesis has been studied in various theoretical models (Kelly and LeRoy, 2005; Acharya and Yorulmazer, 2007; Allen and Gale, 2007; Adrian and Shin, 2010; Shleifer and Vishny, 2010). Additionally, a recent empirical literature indicates different systemic risk measures based on banks’ market capitalization.

In this context, we focus on the loss generated by the reduction in the market equity of bank i at moment t $\left( {Market Equity}_{t}^{i} \right)$ under extreme events and determine at a weekly frequency (i.e., using the last observation of the week) the return of bank i’s market capitalization $\left( R_{Market Equity,t}^{i} \right)$ and the return of the system’s market capitalization $\left( R_{Market Equity,t}^{sys} \right)$. $R_{Market Equity,t}^{sys}=\sum_{i} {\omega_{t}^{i}R}_{Market Equity,t}^{i}$, where $\omega_{t}^{i}$ represents bank i’s weight in the system computed based on weekly market capitalization.^[[1]](#footnote-1)^ Detailed formulae are given in [Table 1](#tab1). The reason for focusing on weekly instead of daily data is that the estimates are more robust in the presence of noise in market capitalization returns. The weekly data on market capitalization are extracted from Datastream.

Based on these returns, we quantify the *Value at Risk* indicator, which expresses the maximum possible loss (as a percent of the total market equity) that bank i or the system could register for a given confidence level α (i.e., 1%) over a specific period. Technically, this loss is found in the left tail of the returns’ distribution function of the market capitalization and involves the estimation of the following loss functions:

|  | ${Prob(R}_{Market Equity,t}^{i}\leq{VaR}_{t}^{i})= \alpha$ | (A4.1) |
| --- | --- | --- |
|  | ${Prob(R}_{Market Equity,t}^{sys}\leq{VaR}_{t}^{sys})= \alpha$ | (A4.2) |

Using the *VaR* and the expected shortfall, we compute banks’ systemic importance through two methodologies that have received notable attention in the literature: *Marginal Expected Shortfall (MES)* and *Conditional Value at Risk (CoVaR)*.

**Online Appendix 4.1. Marginal Expected Shortfall**

To compute the *MES,* we estimate the expected loss of banks’ market equity returns conditional on the system’s market equity returns exceeding the *VaR* limits. ^[[2]](#footnote-2)^ We start by determining the conditional *Expected Shortfall (ES)* of the system’s returns as follows:

|  | ${ES}_{t}^{sys}=E\left[ R_{Market Equity,t}^{sys}\vert R_{Market Equity,t}^{sys}\leq{VaR}_{t}^{sys} \right]$ | (A4.3) |
| --- | --- | --- |

The marginal contribution of a bank to systemic risk corresponds to the partial derivative of the system’s *ES* with respect to bank i’s weight within the system ($\omega_{t}^{i}$):

|  | ${MES}_{t}^{i}=E\left[ R_{Market Equity,t}^{i}\vert R_{Market Equity,t}^{sys}\leq{VaR}_{t}^{sys} \right]=\frac{\partial{ES}_{t}^{sys}}{\partial\omega_{t}^{i}}$ | (A4.4) |
| --- | --- | --- |

To estimate the values of *MES,* we use a multivariate *GARCH-DCC* specification. Following Brownlees and Engle (2017), we consider the joint distribution F of bank i’s and of the system’s market returns:

|  | $\binom{R_{Market Equity,t}^{i}}{R_{Market Equity,t}^{sys}}\vert F_{t-1}\sim N\left( 0,\left( {\sigma_{i,t}^{2} \atop\rho_{i,sys,t}\sigma_{sys,t}\sigma_{i,t}}{\rho_{i,sys,t}\sigma_{sys,t}\sigma_{i,t} \atop\sigma_{sys,t}^{2}} \right) \right)$ | (A4.5) |
| --- | --- | --- |

MES is expressed as the conditional volatility of bank i’s market equity returns and the conditional correlation between bank i’s returns and the system’s returns as follows:

|  | ${MES}_{t}^{i}=E\left[ R_{Market Equity,t}^{i}\vert R_{Market Equity,t}^{sys}<c \right]=E\left[ \sigma_{i,t}\rho_{i,sys,t}\varepsilon_{sys,t}\vert\varepsilon_{sys,t}<c/\sigma_{sys,t} \right]=\sigma_{i,t}\rho_{i,sys,t}E\left[ \varepsilon_{sys,t}\vert\varepsilon_{sys,t}<c/\sigma_{sys,t} \right]$ | (A4.6) |
| --- | --- | --- |

where *c* is equal to the conditional VaR of the system’s market equity returns. We estimate the conditional volatility using a *TARCH* specification and the conditional correlation through a *DCC* model.

**Online Appendix 4.2.** **Conditional Value at Risk**

*Conditional Value at Risk* involves the estimation of the *α* quantile (1%) of the system’s returns distribution over a given period of time conditioned on the event that each bank registers the maximum possible loss:

|  | ${Prob(R}_{Market Equity,t}^{sys}\leq{CoVaR}_{t}^{sys\vert R_{Market Equity,t}^{i}={VaR}_{t}^{i}}\left\vert R_{Market Equity,t}^{i}={VaR}_{t}^{i} \right)= \alpha$ | (A4.7) |
| --- | --- | --- |

We use the *Quantile Regression* method (*QR)* developed by Koenker and Bassett (1978) to estimate each bank’s individual risk and contribution to systemic risk.^[[3]](#footnote-3)^ In comparison with the *Ordinary Least Squares* method, the *QR* method permits the estimation of the dependent variable’s quantiles conditioned on the explanatory variables, being more robust in the presence of extreme market variations.^[[4]](#footnote-4)^ To correct for heteroskedasticity, we apply the *QR* method with robust standard errors.^[[5]](#footnote-5)^

Due to continuous changes in the market environment, both idiosyncratic and systemic risks vary over time, depending on different factors that affect the banking system. To capture this time variation in banks’ risk, we estimate *VaR* and *CoVaR* on a weekly basis, conditioned on several market indices $\boldsymbol{MI}_{t}^{'}$*=(MI_1,t_,…,MI_k,t_)* that incorporate information representative of European financial markets. The choice of these explanatory variables is in agreement with the evidence provided by the empirical literature (Gauthier, Lehar and Souissi, 2012; Adrian and Brunnermeier, 2016). In comparison with the data in these studies, our dataset focuses on a set of factors specific to the European banking market (López-Espinosa et al., 2012; Bostandzic et al., 2020).

As a result of increased counterparty risk, the European interbank markets experienced large interest spreads and jumps in term interest rates after the Lehman collapse. Even banks with a good quality loans portfolio had to borrow at high spreads in the term market due to precautionary liquidity incentives (Acharya and Skeie, 2011). To account for the impact of short-term funding liquidity risk, we employ the spread between the Euribor three-month interbank rate and the euro area government bonds three-month yield curve from the ECB. The long-term government bond yields reached especially high levels in the aftermath of the 2008 financial crisis. To reflect their evolution, we use the change in the Euro AAA government bond yield curve instantaneous forward rate ten years ahead against the one-month residual maturity from the ECB. Additionally, we consider the evolution of real estate and capital markets by employing the real estate price index for Europe from Datastream and the implied volatility index within the Eurozone (VSTOXX) from Bloomberg.^[[6]](#footnote-6)^ A detailed description of these indices is given in Table 1.

Each bank’s idiosyncratic risk is estimated using a linear model that captures the dependence of the bank’s market capitalization returns on market indices lagged one period:

|  | $R_{Market Equity,t}^{i}=\alpha^{i} + \boldsymbol{MI}_{t-1}^{'}\times\boldsymbol{\beta}^{i}+\varepsilon^{i}$ | (A4.8) |
| --- | --- | --- |

Unobserved characteristics of bank i are captured by *α^i^*. $\boldsymbol{MI}_{t-1}^{'}$ is a (1×k) vector of market indices with observations at t-1. $\boldsymbol{\beta}^{i}$ is a (k×1) vector of coefficients that captures bank i’s return dependence relation with the market indices. $\varepsilon_{t}^{i}$ is an *iid* error term.

The return of the system’s market capitalization can change with each bank’s return and with the lagged market indices, following the linear relation from below:

|  | $R_{Market Equity,t}^{sys}=\alpha^{sys\vert i} +{\delta^{sys\vert i}\times R}_{Market Equity, t}^{i} + \boldsymbol{MI}_{t-1}^{'}\times\boldsymbol{\beta}^{sys\vert i}+ \varepsilon_{t}^{sys\vert i}$ | (A4.9) |
| --- | --- | --- |

where $\alpha^{sys|i}$ captures the banking system characteristics conditioned on bank i. $\boldsymbol{\beta}^{sys|i}$ is a (k×1) vector of coefficients that capture the system’s return dependence relation with the one-week lagged market indices $\boldsymbol{MI}_{t-1}^{'}$ conditioned on bank i. $\delta^{sys|i}$ reflects the conditional dependence of the system’s return on bank i’s return, a large coefficient being associated with an enhanced contribution of that bank to systemic risk. $\varepsilon_{t}^{sys|i}$ is an *iid* error term.

Running the *QR* technique on equations (A4.8) and (A4.9) for the 1^st^ quantile and for the median, we obtain the values of the regressors used to calculate each bank’s weekly *VaR* and *CoVaR* in stressed periods (1^st^ quantile) and in normal periods (median):

|  | $\hat{VaR}_{t}^{i}=\hat{\alpha}^{i} + \boldsymbol{MI}_{t-1}^{'}\times{\hat{\boldsymbol{\beta}}}^{i}$ | (A4.10) |
| --- | --- | --- |
|  | $\hat{CoVaR}_{t}^{sys\vert i}=\hat{\alpha}^{sys\vert i} +\hat{\delta}^{sys\vert i}\times\hat{VaR}_{t}^{i}+\boldsymbol{MI}_{t-1}^{'}\times{\hat{\boldsymbol{\beta}}}^{sys\vert i}$ | (A4.11) |

Finally, each bank’s contribution to systemic risk *(CoVaR)* is determined as the difference between *VaR* of the whole system conditioned on the event that the bank reaches the lowest return at a given confidence level (α is 1%) and *VaR* of the whole system conditioned on the event that the bank reaches the median return:

|  | ${\Delta CoVaR}_{t}^{sys\vert i}={CoVaR}_{t}^{sys\vert R_{Market Equity,t}^{i}={VaR}_{\alpha,t}^{i}}-{CoVaR}_{t}^{sys\vert R_{Market Equity,t}^{i}={VaR}_{50\%, t}^{i}}$ | (A4.12) |
| --- | --- | --- |

**References**

Acharya VV, Skeie DR (2011) A model of liquidity hoarding and term premia in inter-bank markets. J Monet Econ 58(5):436–447

Adrian T, Shin HS (2010) Liquidity and leverage. J Financ Intermed 19(3):418–437

Allen F, Gale D (2007) Understanding financial crises. Clarendon Lecture Series in Finance. Oxford University Press, Oxford

Chernozhukov V, Umantsev L (2001) Conditional Value-at-Risk: Aspects of modeling and estimation. Empir Econ 26(1):271–292

Engle RF, Manganelli S (2004) CAViaR: Conditional autoregressive Value at Risk by regression quantiles. J Bus Econ Stat 22(4):367–381

Gauthier C, Lehar A, Souissi M (2012) Macroprudential capital requirements and systemic risk. J Financ Intermed 21(4):594–618

Kelly DL, LeRoy SF (2005) Liquidity and fire sales. In: Orphanides A, Faust J, Reifschneider D (eds) Models and monetary policy: research in the tradition of Dale Henderson, Richard Porter, and Peter Tinsley. Board of Governors of the Federal Reserve System, Washington, DC, pp 249–270

Koenker R, Bassett GS (1978) Regression quantiles. Econometrica 46(1):33–50

Machado JAF, Santos Silva JMC (2013) Quantile regression and heteroskedasticity. Working paper. Available online at <https://jmcss.som.surrey.ac.uk/JM_JSS.pdf>

Portnoy S, Koenker R (1997) The Gaussian hare and the Laplacian tortoise: Computability of squared-error versus absolute-error estimators with discussion. Stat Sci 12(4):279–300

Shleifer A, Vishny RW (2010) Asset fire sales and credit easing. Am Econ Rev 100(2):46–50

1. In an alternative exercise, we define the system by the Euro Stoxx Financial Services Index. Our empirical findings remain robust. [↑](#footnote-ref-1)
2. Technically, this loss is found in the left tail of the returns’ distribution function of the market capitalization and involves the estimation of the next loss function: ${Prob(R}_{Market Equity,t}^{sys}\leq{VaR}_{t}^{sys})= \alpha$. [↑](#footnote-ref-2)
3. Its usage in VaR estimations was initiated by Engle and Manganelli (1999), followed by Chernozhukov and Umantsev (2001). The estimation is done by minimizing the asymmetrically weighted sum of absolute errors with the number of observations corresponding to the quantile of interest. This can efficiently be solved through the algorithm proposed by Portnoy and Koenker (1997), which has proven to be robust in both large and small samples. [↑](#footnote-ref-3)
4. The mean of the dependent variable conditioned on the regressors does not capture all the information necessary to analyze the behavior of the regressand distribution. Therefore, the OLS method is not adequate when the series present extreme values, as it fails to account for the different levels of asymmetry and kurtosis. [↑](#footnote-ref-4)
5. This approach permits the standard errors to be asymptotically valid in the presence of heteroskedasticity and misspecification (Machado and Santos Silva, 2013). [↑](#footnote-ref-5)
6. The market variables are transformed into percentage differences to assure their stationary behavior, as indicated by the unit root tests. [Table 1](#tab1) gives the transformation formulae. [↑](#footnote-ref-6)
